# Phase 2 study of palmitoylethanolamide combined with luteoline in frontotemporal dementia patients

**DOI:** 10.1093/braincomms/fcaf080

**Published:** 2025-03-05

**Authors:** Martina Assogna, Francesco Di Lorenzo, Sonia Bonnì, Ilaria Borghi, Emanuele Cerulli Irelli, Lucia Mencarelli, Michele Maiella, Marilena Minei, Romina Esposito, Elias P Casula, Valentina Pezzopane, Alessia D’Acunto, Francesco Porrazzini, Francesca Candeo, Matteo Ferraresi, Caterina Motta, Clarissa Ferrari, Carlo Caltagirone, Alessandro Martorana, Giacomo Koch

**Affiliations:** Department of Clinical and Behavioural Neurology, Santa Lucia Foundation IRCCS, Rome 00179, Italy; Department of Neurosciences, S. Camillo-Forlanini Hospital, Rome 00152, Italy; Department of Clinical and Behavioural Neurology, Santa Lucia Foundation IRCCS, Rome 00179, Italy; Department of Clinical and Behavioural Neurology, Santa Lucia Foundation IRCCS, Rome 00179, Italy; Department of Clinical and Behavioural Neurology, Santa Lucia Foundation IRCCS, Rome 00179, Italy; Department of Neuroscience and Rehabilitation, University of Ferrara, and Center for Translational Neurophysiology of Speech and Communication (CTNSC), Italian Institute of Technology (IIT), Ferrara 44121, Italy; Department of Human Neurosciences, Sapienza, University of Rome, Rome 00185, Italy; Department of Clinical and Behavioural Neurology, Santa Lucia Foundation IRCCS, Rome 00179, Italy; Department of Clinical and Behavioural Neurology, Santa Lucia Foundation IRCCS, Rome 00179, Italy; Department of Clinical and Behavioural Neurology, Santa Lucia Foundation IRCCS, Rome 00179, Italy; Department of Clinical and Behavioural Neurology, Santa Lucia Foundation IRCCS, Rome 00179, Italy; Department of Clinical and Behavioural Neurology, Santa Lucia Foundation IRCCS, Rome 00179, Italy; Department of Systems Medicine, University of Tor Vergata, Rome 00133, Italy; Department of Clinical and Behavioural Neurology, Santa Lucia Foundation IRCCS, Rome 00179, Italy; Department of Neuroscience and Rehabilitation, University of Ferrara, and Center for Translational Neurophysiology of Speech and Communication (CTNSC), Italian Institute of Technology (IIT), Ferrara 44121, Italy; Department of Clinical and Behavioural Neurology, Santa Lucia Foundation IRCCS, Rome 00179, Italy; Department of Clinical and Behavioural Neurology, Santa Lucia Foundation IRCCS, Rome 00179, Italy; Department of Clinical and Behavioural Neurology, Santa Lucia Foundation IRCCS, Rome 00179, Italy; Department of Clinical and Behavioural Neurology, Santa Lucia Foundation IRCCS, Rome 00179, Italy; Department of Systems Medicine, University of Tor Vergata, Rome 00133, Italy; Office of Research and Clinical Trials, Fondazione Poliambulanza Istituto Ospedaliero, Brescia 25124, Italy; Department of Clinical and Behavioural Neurology, Santa Lucia Foundation IRCCS, Rome 00179, Italy; Department of Systems Medicine, University of Tor Vergata, Rome 00133, Italy; Department of Clinical and Behavioural Neurology, Santa Lucia Foundation IRCCS, Rome 00179, Italy; Department of Neuroscience and Rehabilitation, University of Ferrara, and Center for Translational Neurophysiology of Speech and Communication (CTNSC), Italian Institute of Technology (IIT), Ferrara 44121, Italy

**Keywords:** frontotemporal dementia, neuroinflammation, palmitoylethanolamide, synaptic activity, cognitive decline

## Abstract

Frontotemporal dementia is a devastating neurodegenerative disorder for which no pharmacological treatments have been approved. Neuroinflammation plays a central role in driving the pathogenic mechanisms underlying frontotemporal dementia. In the last few years, co-ultramicronized palmitoylethanolamide combined with luteoline has emerged as a potential therapeutic molecule in neurodegenerative disorders pathogenically related to frontotemporal dementia, for its demonstrated strong anti-inflammatory and neuroprotective properties. Here we wanted to determine whether treatment with co-ultramicronized palmitoylethanolamide combined with luteoline may have a clinical impact in frontotemporal dementia patients. We performed a Phase 2, monocentric, randomized, double-blind, placebo-controlled trial to evaluate the safety and efficacy of co-ultramicronized palmitoylethanolamide combined with luteoline in frontotemporal dementia patients. Forty eight patients with a diagnosis of probable frontotemporal dementia were randomly assign in a 1:1 ratio to receive co-ultramicronized palmitoylethanolamide combined with luteoline oral suspension at the dosage of 700 mg + 70 mg twice/day (*n* = 25) or placebo twice/day (*n* = 23) for 24 weeks. The primary efficacy outcome measure was the change at 24-weeks in the Clinical Dementia Rating Dementia Staging Instrument from the National Alzheimer’s Coordinating Center and frontotemporal lobar degeneration modules—sum of boxes (CDR plus NACC FTLD—SoB). Secondary outcome measures included the Frontal Assessment Battery, Screening for Aphasia in Neurodegeneration, Alzheimer’s Disease Cooperative Study—Activities of Daily Living, Neuropsychiatric Inventory, Mini-Mental State Examination and Addenbrooke’s Cognitive Examination Revised. Among 48 patients randomized [mean (SD) age 63.2 (8.4), 23 (47.9%) female], 45 (93%) completed the study. Patients in the co-ultramicronized palmitoylethanolamide combined with luteoline group showed less decline for the primary outcome measure (CDR plus NACC FTLD) as compared with patients treated with placebo. The estimated mean change (W0–W24) in CDR plus NACC FTLD score was 0.53 for the co-ultramicronized palmitoylethanolamide combined with luteoline group [95% confidence interval (0.12–0.94)] and 1.39 for the placebo group [95% confidence interval (0.96–1.82)], with an estimated mean difference between of 0.86 [95% confidence interval (0.28–1.45), *P* = 0.005]. Estimated mean change in Alzheimer’s Disease Cooperative Study—Activities of Daily Living score was −1.8 for co-ultramicronized palmitoylethanolamide combined with luteoline (95% confidence interval, −3.67 to 0.06) and −7.39 for placebo (95% confidence interval −9.34 to −5.45). Estimated mean change in screening for Aphasia in neurodegeneration scores was −3.987 for co-ultramicronized palmitoylethanolamide combined with luteoline (95% confidence interval, −7.75 to −0.22) and −10.35 for placebo (95% confidence interval, −14.33 to −6.37). No effect of treatment was found on other secondary outcome measures. Our results demonstrate that co-ultramicronized palmitoylethanolamide combined with luteoline shows promising efficacy in slowing down the progression of cognitive and functional symptoms in frontotemporal dementia patients. These findings warrant further investigation and offer potential for the development of effective therapeutic strategies for frontotemporal dementia.

## Teaser

Assogna *et al*. report the results of a Phase 2, randomized, double-blind, placebo-controlled trial in 48 patients with a diagnosis of probable frontotemporal dementia, showing that co-ultramicronized palmitoylethanolamide combined with luteoline exhibits encouraging efficacy in slowing down the progression of cognitive and functional symptoms.

## Graphical Abstract

**Graphical Abstract:**
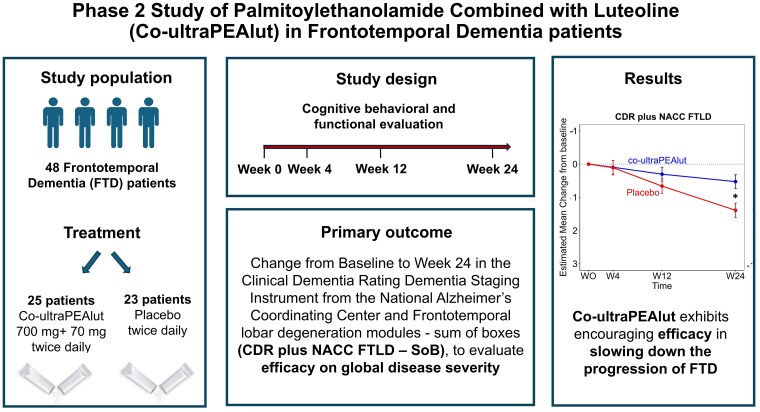

## Introduction

Frontotemporal dementia (FTD) is an harmful neurodegenerative disorder, primarily affecting the frontal and/or temporal lobes, representing one of the leading causes of presenile neurodegenerative dementia.^1^ FTD is a heterogeneous disorder from a clinical, pathological and genetic perspective.^2^ From a clinical viewpoint, FTD is characterized by impairment of frontal executive functions, language deficits or changes in behaviour and personality. Depending on the relative principal cognitive and behavioural symptoms, three main clinical syndromes have been identified, namely the behavioural variant of FTD (bvFTD), the non-fluent/agrammatic variant of primary progressive aphasia and the semantic variant of PPA (svPPA).^3-5^ Progression of deficits differ within individuals and phenotypes, leading to substantial life expectancy reduction, with a profound decline in global functioning, increased caregiver dependency and death due to complications.^6^ Currently, there is no effective disease-specific pharmacological treatment effective in reducing the progression of FTD. Therapeutic strategies are mostly based on the off-label use of symptomatic agents to manage behavioural symptoms with little evidence from randomized, placebo-controlled trials.^7,8^

Recent findings support a pivotal role of neuroinflammation in driving the pathogenic mechanisms underlying FTD since the early phases of the disease.^9,10^ It has been hypothesized that novel drugs targeting and modulating neuroinflammation could potentially slow down the progression of the disease.^11^ On the other hand, FTD is also characterized by synaptic dysfunction and neurotransmitter deficits, that have been reported widely in animal models and in FTD patients.^12^ Neuroinflammation and microglial alterations contribute to synaptic dysfunction and neurodegeneration process.^13,14^ In the last few years a new composite, which is a formulation of palmythoilethanolamide (PEA), a saturated *N*-acylethanolamide belonging to the family of endocannabinoids and the antioxidant flavonoid luteolin (Lut) subjected to an ultramicronization process [co-ultramicronized palmitoylethanolamide combined with luteoline (co-ultraPEAlut)], has emerged as a potential therapeutic molecule in neurodegenerative disorders for its strong anti-inflammatory and neuroprotective properties, as shown in pathogenical conditions related to FTD, such as amyotrophic lateral sclerosis.^15^ Moreover, PEA modulates synaptic activity, mostly by enhancing GABAergic neurotransmission, which is impaired in patients with FTD, through the activation of cannabinoid receptor type 1 (CB1) receptor at a presynaptic site.^16,17^ In a recent pilot study from our group,^18^ we found that co-ultraPEAlut was able to improve cognitive functions and neuropsychiatric disturbances by increasing GABAergic activity and high-frequency cortical oscillatory activity on the prefrontal cortex. Hence, we performed a Phase 2, randomized, placebo-controlled trial study performed to evaluate the safety and efficacy of co-ultraPEAlut in FTD patients.

## Materials and methods

### Patients and study design

This was a Phase 2, monocentric, randomized, double-blind, placebo-controlled trial conducted at Santa Lucia Foundation IRCCS in Rome, Italy, from June 2019 to December 2022, in patients with a diagnosis of a probable FTD.^3,4^ The trial was approved by the review board and the local Ethics Committee (Ce/PROG.739) in accordance with the principles of the Declaration of Helsinki and the International Conference on Harmonization Good Clinical. All the participants or their relatives or legal representatives provided written informed consent before screening. Patients could withdraw at any point during the study without prejudice. This report followed the CONSORT reporting guideline for randomized studies. The PEA-FTD study was registered on the clinicaltrial.gov website (NCT04489017). The trial was conducted in collaboration with Epitech Group pharmaceuticals, which provided the active drug and placebo. An independent committee continuously monitored the patients’ safety according to the Data Monitoring Committee Charter. The trial protocol can be found in Supplementary Section 1. Patients were eligible if they: (i) had an established diagnosis of probable FTD based on current clinical criteria;^3,4^ (ii) were aged from 40 to 85 years; (iii) they had a Clinical Dementia Rating-FTD (CDR-FTD) total score of ≤2 at Screening; (iv) had not received treatment with acetylcholinesterase inhibitor, i.e. donepezil, galantamine, or rivastigmine, at the time of screening; and (v) were able to comply with the study procedures according to the investigator’s assessment. In all cases, the diagnosis was supported by structural brain imaging including evidence of frontotemporal hypometabolism at 18-FDG-PET/TC imaging and cerebrospinal fluid Aβ1-42 dosage or amyloid PET imaging to rule out Alzheimer’s disease pathology. Briefly, levels of Aβ 1-42, t-tau and p-tau phosphorylated concentrations were determined using a sandwich enzyme-linked immunosorbent assay (EUROIMMUN ELISA^©^). The cut-off values used to define a CSF Alzheimer’s disease-like profile, determined following Euroimmun guidelines, were CSF Aβ42 < 600 pg/ml, CSF p-tau > 65 pg/ml, CSF t-tau > 450 pg/ml. PET amyloid imaging was acquired using 370 MBq (10 mCi) of [18F]-florbetapir or [18F]-flutemetamol and visual readings were performed by nuclear medicine physicians who were blinded to the patients’ diagnosis, following the procedures provided by the ligand manufacturer. Patients were excluded if they had significant neurodegenerative disorders of the CNS other than FTD, significant intracranial focal or vascular pathology seen on brain MRI scan or history of stroke and if they had been treated within 3 months before baseline with antipsychotics, anti-seizure and antidepressants drugs. Before entering the trial, all patients with a family history of cognitive impairment and/or presenile onset of FTD underwent genetic testing for GRN, C9orf72, MAPT P301L and TARDBP gene mutations.

### Randomization and masking

After recruitment and baseline evaluations eligible participants were randomly assigned in a 1:1 ratio to receive co-ultraPEAlut oral suspension at the dosage of 700 mg + 70 mg or placebo twice daily (one sachet of oral suspension in the morning and one in the evening) for 24 weeks. A simple randomization scheme was performed and assigned by an independent statistician. The dose of co-ultraPEAlut used in this trial was chosen based on the results of a previous pilot study in FTD patients conducted by our group.^18^ The treatment was administered for 24 weeks with no interruptions. Clinic visits or video-telephone contacts (during COVID-19 lockdown) were conducted at baseline, at 4, 12 and 24 weeks. FTD patients were enrolled by expert neurologists (A.M., F.D.L., C.M., G.K. and M.A.) at Memory Clinics of Santa Lucia Foundation IRCCS and Tor Vergata University, Rome, Italy. Cognitive and behavioural evaluations were performed at Santa Lucia Foundation by expert neuropsychologists (I.B., S.B. and Ma.M.). All the raters and investigators were blinded to the treatment allocation. Safety assessment was done through the recording of adverse events. Vital signs documentation, physical and neurological examination were also performed at each study visit (or upon early termination). Treatment adherence was assessed at each study visit throughout the study as the amount of treatment returned used or unused.

### Outcome measures

The primary efficacy outcome measure was the change at 24-weeks from baseline in the CDR Dementia Staging Instrument plus behaviour and language domains from the National Alzheimer’s Coordinating Center and frontotemporal lobar degeneration modules—sum of boxes (CDR plus NACC FTLD—SoB), a scale measuring global disease severity, specific for FTLD patients.^19^ The CDR plus NACC FTLD—SoB consists in the evaluation of eight cognitive and functional domains, which include memory, orientation, judgment and problem-solving, community affairs, home and hobbies, personal care, behaviour/personality and language, with each category rated on a five-point scale, except the item personal care which is rated on a four-point scale. The total of the domain ratings corresponds to the CDR Sob; scores range from 0 to 24, with higher scores meaning higher disease severity. As meticulously recorded on clinicaltrial.gov website (NCT04489017) and in the Trial Protocol, a modification in the pre-specified primary end-point occurred following the initiation of the trial, based on upcoming literature findings.^20,21^ Indeed, emerging evidence suggests that, for trials potentially involving patients with any of the FTD syndromes, priority should be given to functional and global composite measures. These measures take into account the diverse manifestations of these disorders and are consider more attractive clinical outcome metrics due to their ability to yield comparable sample size estimates across all groups and their inherent clinical significance.^20,21^ The decision to prioritize CDR plus NACC FTLD—SoB was made by the Principal Investigator (G.K.) independently of trial data and prior to unblinding.

The secondary outcome measure included the change at 24 weeks from baseline of the frontal assessment battery (FAB, a battery to evaluate executive functions, scores range from 0 to 18, with higher scores indicating better frontal cognitive function),^22,23^ the screening for aphasia in neurodegeneration (SAND, Battery to evaluate language functions, scores range from 0 to 84 with a higher score meaning less severe language deficits),^24^ Alzheimer’s Disease Cooperative Study—Activities of Daily Living (ADCS-ADL, scale to evaluate activities of daily living; scores range from 0 to 78, with higher scores indicating worse function),^25^ neuropsychiatric inventory scale (NPI, scale to assess behavioural changes, the scores range from 0 to 144 with a higher score meaning more severe behavioural disturbances),^26^ Mini-Mental State Examination (MMSE, a battery to evaluate global cognition, with scores range from 0 to 30 with a higher score meaning less cognitive impairment),^27^ Addenbrooke’s Cognitive Examination Revised (ACE-R, a battery to evaluate global cognition, scores range from 0 to 100 with a higher score meaning less cognitive impairment),^28^ the frontal behavioural inventory (FBI, a scale to assess behavioural changes, scores range from 0 to 72 with a higher score meaning more severe behavioural disturbances).^29^

### Statistical analysis

The appropriateness of our sample size, i.e. 48 randomly assigned patients (25 to co-ultraPEAlut treatment and 23 to placebo), was based on the effect size obtained in a preliminary study of our group using a similar protocol.^18^ In this study, we obtained a medium effect size of 0.93. With this effect size, adopting a two-tailed paired Wilcoxon signed-rank test, with type I error alpha = 0.05, the minimum sample for reaching a power (1 − β) of 0.95 was estimated equal to *n* = 18 patients per group. We decided to increase our sample size by at least 30% considering possible drop-outs.

Standard descriptive statistics were employed to describe the study sample and the distribution of outcome measures. The primary outcome was analysed in the intention to treat (ITT) population, which consisted of all randomized patients who received at least one dose of the study drug or placebo. We used a repeated measures linear mixed model with participant as random effect, and treatment, time, FTD variant and age as fixed factors. Missing data were handled with weighted estimating equations for longitudinal data. As sensitivity analysis, the primary outcome was analysed through the same repeated measures linear mixed model using complete data analysis. An additional sensitivity analysis was performed to evaluate treatment effects according to FTD subgroups, performing two distinct repeated measures linear mixed models in PPA and bvFTD patients, respectively. Secondary outcomes except for NPI were analysed using a repeated measures linear mixed model with the same covariates used for the primary outcome. NPI was analysed using a generalized linear mixed model assuming a Poisson distribution. An unstructured covariance matrix was used for both primary and secondary outcome analyses. The confidence interval (CI) widths of secondary analyses have not been adjusted to account for multiple testing and should not be used to draw definitive conclusions about treatment effects. All analyses were performed with the use of RStudio software, version 4.2.3.

### Data availability

Deidentified data collected in the present study will be available to qualified investigators upon request as long as data transfer agrees with EU Legislation on the general data protection regulation. Data transfer will be regulated by material transfer agreements and should be authorized by institutional Review Boards.

## Results

**Figure 1:**
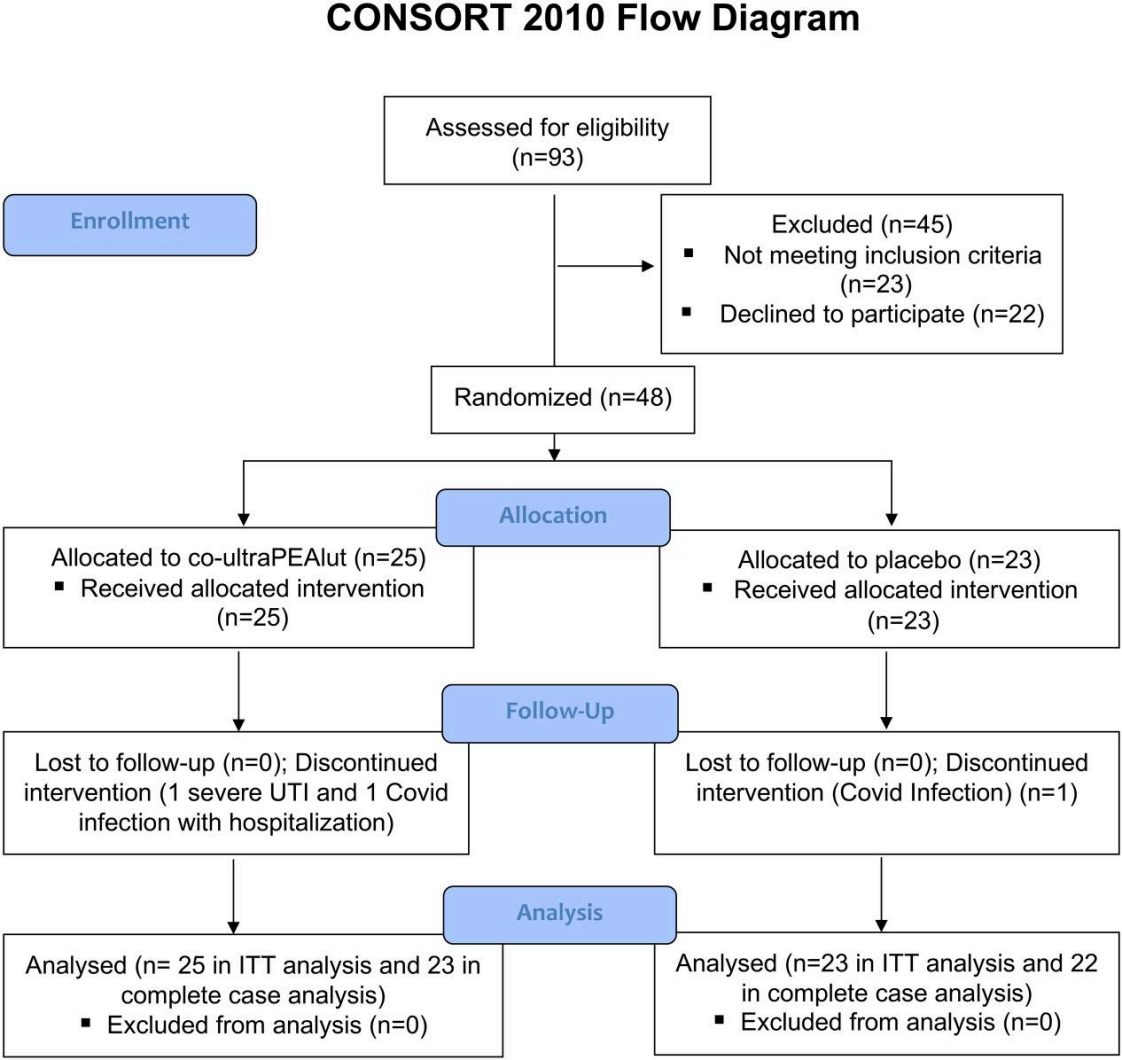
**Study flowchart. Randomization, trial-group assignment and follow-up in the trial.** Co-ultraPEAlut, co-ultramicronized palmitoylethanolamide combined with luteoline; ITT, intention-to-treat analysis; UTI, urinary tract infection.

A total of 93 patients were screened, of which 48 were randomized between June 2019 and May 2022 to the trial group: 25 to co-ultraPEAlut treatment and 23 to placebo (Fig. 1). FTD patients’ demographic and baseline clinical characteristics are summarized in Table 1. The mean age of patients was 63.2 ± 8.4 of which 23 (47.9%) were female. In the PPA group, all patients had a diagnosis of nfvPPA except two patients that were diagnosed with svPPA (both in the placebo group). Three patients (one co-ultraPEAlut and two placebo) had evidence of a genetic form of FTD (C9orf72 mutation), whereas no mutation was identified in the GRN, MAPT or TARDBP genes. One patient in each treatment group had a diagnosis of a comorbid autoimmune disease (one with psoriasis and Hashimoto’s thyroiditis and one with Hashimoto’s thyroiditis). None of the patients were on stable treatment with anti-inflammatory drugs or therapies. In total 3 patients withdrew from the trial before completion, of which 2 were assigned to the co-ultraPEAlut group and 1 to the placebo group, so that a total of 45 patients (93%) completed the treatment period. Treatment adherence was high in both the placebo group (90.6%) and in the co-ultraPEAlut group (91.4%). In the current trial, adverse events were similar between the two groups and are reported in Table 2. In the co-ultraPEAlut group, one patient reported a severe urinary tract infection and another got a COVID-19 infection with severe pneumonia, while in the placebo group one patient got a severe COVID-19 infection and declined to continue. Moreover, in the co-ultraPEAlut group, two patients reported headache, two nausea and one dizziness, while in the placebo group three patients reported headache, one nausea and one diarrhoea. Throughout the trial no patient was unblinded and no minor symptoms capable of potentially unblinding participants were observed or documented. No significant other adverse events were reported. Patients from both groups were included in the ITT analyses. During the Italian lockdown for the COVID-19 pandemic (9 March 2020–18 May 2020 and from 13 October to 26 November 2020) all the patients already enrolled in the study were evaluated by video-telephone contacts. In particular, 5 patients were evaluated by video-telephone contacts at Week 24 (2 in the co-ultraPEAlut and 3 in the placebo group), 11 patients at Week 12 (5 in the co-ultraPEAlut and 6 in the placebo group), while 3 patients were evaluated by video-telephone contacts at Week 4 (1 in the co-ultraPEAlut and 2 in the placebo group).

**Table 1:** Baseline demographics and clinical characteristics of patients

	co-ultraPEAlut (*n* = 25)	Placebo (*n* = 23)	*P*-value
Sex, female	12 (48%)	11 (47.8%)	1
Age, mean (SD), years	66.1 (7.51)	60.1 (8.35)	0.01^a^
Education, mean (SD), years	10.4 (4.1)	12.3 (3)	0.07
Disease duration, mean (SD), years	3.2 (1.8)	3.4 (2.3)	0.79
FTD variant			0.56
PPA, *n* (%)	11 (44)	8 (34.8)	
bvFTD, *n* (%)	14 (56)	15 (65.2)	
CDR plus NACC FTLD, mean (SD)	7.4 (4.9)	8 (3.6)	0.60
MMSE score, mean (SD)	20.8 (7.8)	19.9 (7.5)	0.69
FAB score, mean (SD)	7.5 (4.7)	8.2 (4.5)	0.60
ADCS-ADL score, mean (SD)	52.2 (19.8)	49.2 (13.4)	0.56
NPI score, mean (SD)	24.6 (18.1)	28.5 (18.1)	0.46
SAND score, mean (SD)	54.3 (17.9)	56.7 (17.5)	0.67
FBI score, mean (SD)	19.8 (10.7)	25.6 (11.6)	0.08
ACE-R score, mean (SD)	59.7 (22.9)	58.6 (20.5)	0.88

ACE-R, Addenbrooke’s cognitive examination revised; ADCS-ADL, Alzheimer disease cooperative study activities of daily living scale; bvFTD, behavioural variant frontotemporal dementia; CDR plus NACC FTLD, CDR Dementia Staging Instrument plus behaviour and language domains from the National Alzheimer’s Coordinating Center and frontotemporal lobar degeneration modules—sum of boxes; FAB, frontal assessment battery; FBI, frontal behavioural inventory; FTD, frontotemporal dementia; MMSE, mini-mental state examination; NPI, neuropsychiatric inventory; PPA, primary progressive aphasia; SAND, screening for aphasia in neurodegeneration; SD, standard deviation.

^a^Indicate statically significant variables.

**Table 2:** Summary of adverse events by treatment group

Event	co-ultraPEAlut (*n* = 25)	Placebo (*n* = 23)
Overview of AEs, No (%)		
Death	0 (0)	0 (0)
Death considered related to treatment	0 (0)	0(0)
Participants with >1 serious AE	0 (0)	0 (0)
Treatment discontinuations due to AEs	2 (8)	1 (4.3)
AE		
UTI	1 (4)	0
COVID-19	1 (4)	1 (4.3)
Headache	2 (8)	3 (13.04)
Nausea	2 (8)	1 (4.3)
Diarrhoea	0 (0)	1 (4.3)
Dizziness	1 (4)	0

AEs, adverse events; UTI, urinary tract infection.

### Primary outcome measure

**Figure 2:**
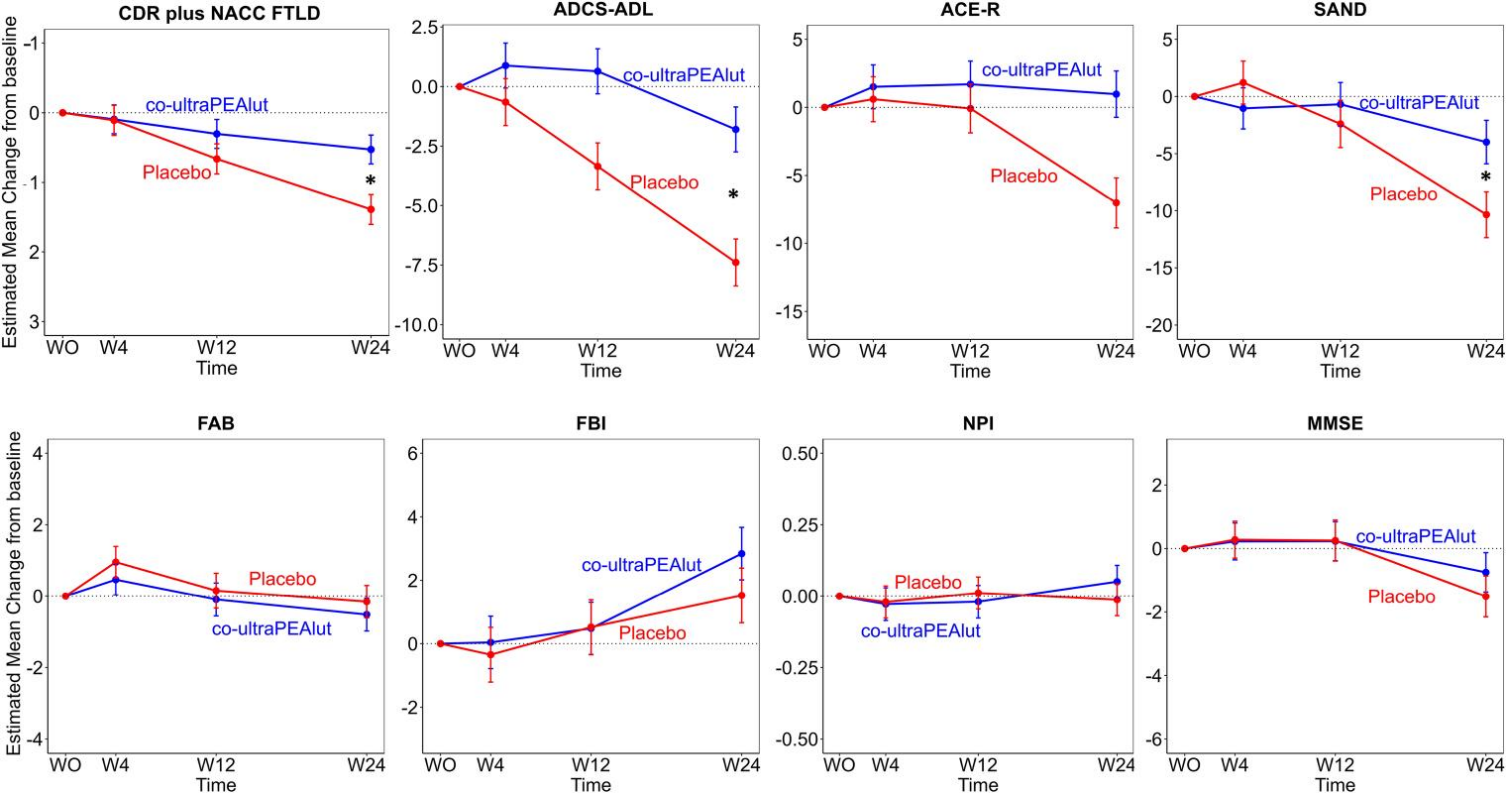
**Clinical data results.** The generalized linear mixed model estimated mean change from baseline is shown for the CDR Dementia Staging Instrument plus behaviour and language domains from the CDR plus NACC FTLD—SoB, with an estimated mean difference between co-ultraPEAlut and placebo of 0.86 [95% CI (0.28–1.45), *P* = 0.005]; for ADCS-ADL, with an estimated mean difference between co-ultraPEAlut and placebo of −5.59 [95% CI (−8.26 to −2.92), *P* < 0.001]; for the ACE-R, with an estimated mean difference between co-ultraPEAlut and placebo of −6.05 [95% CI (−10.95 to −1.15), *P* = 0.02]; for the SAND, with an estimated mean difference between co-ultraPEAlut and placebo of −6.36 [95% CI (−11.77 to −0.95), *P* = 0.02]; for the FAB, with an estimated mean difference between co-ultraPEAlut and placebo of −0.67 [95% CI (−1.94 to 0.60), *P* = 0.30]; for the FBI, with an estimated mean difference between co-ultraPEAlut and placebo of −1.32 [95% CI (−3.66 to 1.02), *P* = 0.27]; for the NPI, with an estimated mean difference between co-ultraPEAlut and placebo of −0.06 [95% CI (−0.22 to 0.09), *P* = 0.43]; for the MMSE, with an estimated mean difference between co-ultraPEAlut and placebo of −0.76 [95% CI (−2.5 to 0.98), *P* = 0.39]. Baseline is plotted at Week 0, which is the mean assessment time of the baseline measurement as offset from the first administration of the cco-ultraPEAlutor placebo. Cognitive evaluations were collected also at 4, 12 and 24 weeks. Error bars indicate standard errors. Reported estimated mean differences between groups were calculated from baseline to 24 weeks. * indicate *P* values <0.05.

There were no differences in the mean baseline CDR plus NACC FTLD total score between the co-ultraPEAlut group (mean = 7.38, SD = 4.94) and the placebo group (mean = 8.04, SD = 3.64). The ITT analysis of the primary outcome showed a significant result in terms of the difference between Time × Group (*P* = 0.012) interaction. Patients in the co-ultraPEAlut group showed less decline in global disease severity (CDR plus NACC FTLD) compared with patients treated with placebo (Fig. 2). The estimated mean change (W0–W24) in CDR plus NACC FTLD score was 0.53 for the co-ultraPEAlut group [95% CI (0.12–0.94)] and 1.39 for the placebo group [95% CI (0.96–1.82)], with an estimated mean difference of 0.86 [95% CI (0.28–1.45), *P* = 0.005]. Sensitivity analysis using complete data analysis provided similar results, with estimated mean difference between co-ultraPEAlut and placebo = 0.72, [95% CI (0.15–2.48), *P* = 0.015].

An additional sensitivity analysis was conducted to account for baseline functional and behavioural severity, including ADCS-ADL and FBI values as additional covariates in the model. This analysis confirmed a significant effect of co-ultraPEA Lut compared with placebo, with an estimated mean difference of 0.71 [95% CI (0.12–1.31), *P* = 0.019].

**Figure 3:**
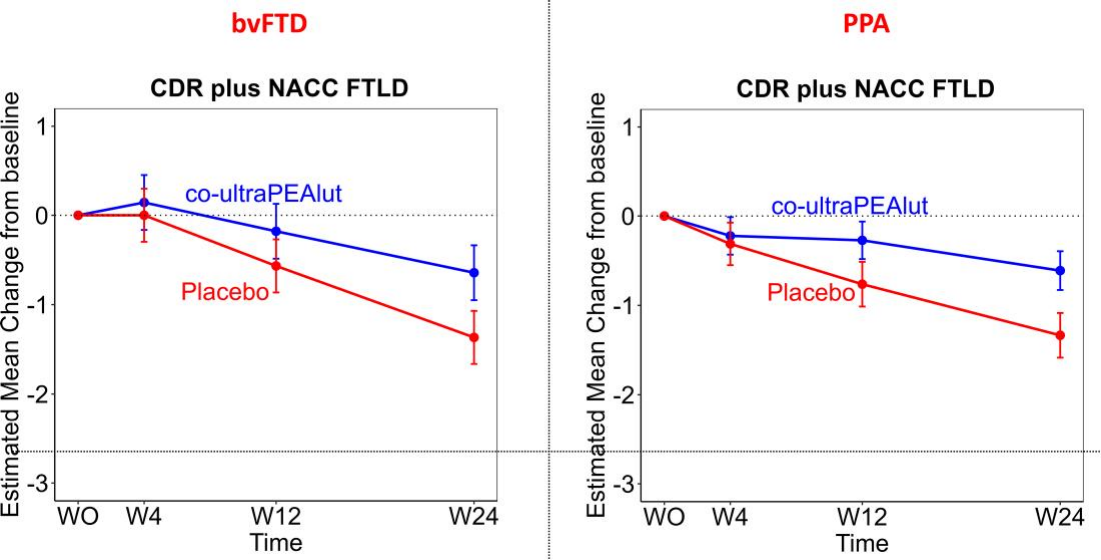
**Sensitivity analysis for the primary outcome in the PPA and bvFTD population.** The generalized linear mixed model estimated mean change from baseline is shown for the primary outcome CDR Dementia Staging Instrument plus behaviour and language domains from the CDR plus NACC FTLD—SoB, with higher scores meaning higher disease severity in the bvFTD and the PPA subgroups. The estimated mean difference between co-ultraPEAlut and placebo among bvFTD patients was 0.72 [95% CI (−0.11 to 1.56), *P* = 0.09]; the estimated mean difference between co-ultraPEAlut and placebo among PPA patients was 0.72, [95% CI (0.07–1.37), *P* = 0.034]. Baseline is plotted at Week 0, which is the mean assessment time of the baseline measurement as offset from the first administration of the co-ultraPEAlut or placebo. Cognitive evaluations were collected also at 4, 12 and 24 weeks. Error bars indicate standard errors. Reported estimated mean differences between groups were calculated from baseline to 24 weeks.

An additional sensitivity analysis was performed to evaluate treatment effects according to FTD subgroups. Such analysis stratified for disease subgroups yielded comparable results. The estimated mean difference between co-ultraPEAlut and placebo among bvFTD patients was 0.72, [95% CI (−0.11 to 1.56), *P* = 0.09]; the estimated mean difference between co-ultraPEAlut and placebo among PPA patients was 0.72, [95% CI (0.07–1.37), *P* = 0.034] (Fig. 3).

### Secondary outcomes

The analysis of the secondary outcomes showed significant differences between the co-ultraPEAlut group and the placebo group for the ADCS-ADL scores and SAND scores (Fig. 2). At baseline ADCS-ADL scores were similar between groups (co-ultraPEAlut mean = 52.17, SD = 19.82; placebo mean = 49.18, SD = 13.43). The estimated mean change in ADCS-ADL score was −1.8 for the co-ultraPEAlut group (95% CI, −3.67 to 0.06) and −7.39 for the placebo group (95% CI −9.34 to −5.45), suggesting less functional decline in the treated group compared with the placebo group (*P* < 0.001 for interaction time × group). Comparable baseline mean SAND scores were found for both groups (co-ultraPEAlut mean = 54.34, SD = 17.95; placebo mean = 56.7, SD = 17.49). The estimated mean change in SAND scores was −3.99 for the co-ultraPEAlut group (95% CI, −7.75 to −0.22) and −10.35 for the placebo group (95% CI, −14.33 to −6.37), showing an advantage of the co-ultraPEAlut treatment compared with placebo in language function (*P* = 0.025 for interaction). The baseline ACE-R mean scores were similar for both groups (co-ultraPEAlut mean = 59.71, SD = 22.94; placebo mean = 58.56, SD = 20.52). The estimated mean change in ACE-R score was −0.97 for the co-ultraPEAlut group (95% CI, −4.34 to 2.40) and −7.02 for the placebo group (95% CI, −10.67 to −3.37), suggesting a trend towards less global cognitive decline at the end of the treatment period in the co-ultraPEAlut group (*P* = 0.101) (Fig. 2). No significant effect of co-ultraPEAlut treatment was found on MMSE, FAB, NPI and FBI scores (Fig. 2). The baseline FAB mean scores were similar for both groups (co-ultraPEAlut = 7.52, SD = 4.68; placebo = 8.23, SD = 4.47). The estimated mean change in FAB score was 0.52 for the co-ultraPEAlut group (95% CI, −0.4 to 1.43) and −0.15 for the placebo group (95% CI, −1.06 to 0.75), which revealed no effects of co-ultraPEAlut treatment on frontal lobe functions (*P* = 0.352). The baseline mean for NPI total score did not differ across groups (co-ultraPEAlut = 24.56, SD = 18.94; placebo = 28.5, SD = 18.05). The estimated mean change in NPI score was 0.05 for the co-ultraPEAlut group (95% CI, −0.06 to 0.16) and −0.01 for the placebo group (95% CI, −0.12 to 0.1), suggesting no significant effects on behavioural symptoms (*P* = 0.679). The FBI mean baseline scores were similar for both groups (co-ultraPEAlut = 19.76, SD = 10.67; placebo = 25.57, SD = 11.58). The estimated mean change in FBI score was 2.84 for the co-ultraPEAlut group (95% CI, 1.21–4.47) and 1.52 for the placebo group (95% CI, −0.18 to 3.22), suggesting no significant effects (*P* = 0.642). Finally, baseline MMSE mean scores were similar across groups (co-ultraPEAlut = 20.78, SD = 7.83; placebo = 19.86, SD = 7.53). The estimated mean change in MMSE score was −0.75 for the co-ultraPEAlut group (95% CI, −1.97 to 0.47) and −1.51 for the placebo group (95% CI, −2.79 to −0.23), suggesting no significant effects (*P* = 0.792).

## Discussion

Overall, the treatment was well tolerated with few mild side effects similar in both groups. This 24-week randomized clinical trial showed that co-ultraPEAlut treatment at the oral dosage of 700 mg + 70 mg twice daily reduced the progression of global disease severity in FTD patients as measured by CDR plus NACC FTLD. The analysis stratified for disease subgroups (PPA versus bvFTD) yielded comparable results. Moreover, comparing the co-ultraPEAlut with the placebo group we observed less decline in the ADCS-ADL scores, suggesting that its use has an additional role in slowing functional impairment. We also observed an advantage of co-ultraPEAlut treatment in language function and a trend towards a less global cognitive decline as revealed by the ACE-R scores. However, no significant effect of co-ultraPEAlut treatment was found on frontal executive functions and behavioural symptoms.

Current pharmacological research in FTD is limited by the heterogeneity of FTLD syndromes, which constitute a challenge in efficiently measuring treatment effects.^30^ In this scenario, the CDR plus NACC FTLD has been identified as the best instrument to detect clinical features and rates of progression across the FTLD spectrum even at early stages in clinical trials.^19,31^ Previous studies have shown that FTD, although with an inter-individual and intra-phenotype variety, brings to a dramatic decline in patients functioning, with loss of independence and lower survival rates compared with Alzheimer’s disease.^32-34^ Actually, there are no reliable approved treatments to treat FTD, with most therapeutic strategies that are based on off-label use of symptomatic agents.

Our findings in FTD patients are consistent with numerous preclinical and clinical studies that highlight ultramicronized PEA (a formulation designed to enhance PEA bioavailability and facilitate its penetration across the blood-brain barrier) as a promising therapeutic agent for various neurodegenerative and neuroinflammatory conditions, including amyotrophic lateral sclerosis and post-COVID neurological syndrome.^35-37^ Specifically, co-ultraPEAlut has been proposed to protect nervous tissue from damage and mitigate GABAergic interneuron dysfunction by reducing mast cell, astrocyte, microglial activation and by limiting the release of pro-inflammatory mediators.^35^

Moreover, PEA can enhance GABAergic transmission downregulating the synthesis of the endocannabinoid 2-AG, which acts retrogradely onto presynaptic CB1 cannabinoid receptors and suppresses GABA release.^38^ PEA can also control GABA transmission, by enhancing indirectly the levels of other endocannabinoids, through the so-called entourage effect.^38^ In addition, PEA can influence other endocannabinoid targets, such as the TRPV1 channel. In animal models, the activation of TRPV1 may reverse memory deficits and improve hippocampal function by restoring gamma oscillations, which are disrupted in conditions such as Alzheimer’s disease and FTD.^39,40^ Furthermore, PEA appears to protect neurons by modulating glutamatergic transmission and synaptic plasticity.^41^ Interestingly, in a transcranial magnetic stimulation study, altered mechanisms of plasticity were observed also in pre-symptomatic FTD carriers with progranulin (GRN) and C9orf72 genes mutation,^42^ reinforcing the notion that alteration of synaptic machinery begins years before the onset of clinical symptoms. PEA can also inhibit glutamate release in nerve terminals, possibly by reducing calcium influx through the activation of presynaptic cannabinoid CB1 receptors. This inhibition of abnormal glutamatergic activity, which was hypothesized to be impaired also in FTD^43^ could offer anti-excitotoxic effects, potentially counteracting neurodegeneration. Hence, we argue that co-ultraPEAlut may be able to restore disrupted neurotransmission and impaired synaptic plasticity trough a modulation of neuroinflammation. Further pre-clinical and early clinical studies are needed to dig into these potential mechanisms of action across the FTD spectrum.

Despite potential bias in the CDR plus NACC FTLD results due to the heterogeneity of FTD variants in our trial, the additional sensitivity analysis stratified for disease subgroups revealed comparable results between bvFTD and PPA patients. Nevertheless, we cannot exclude a possible variant-independent co-ultraPEAlut potential effect on disease progression, warranting confirmation in larger trials. Moreover, our preliminary results are further substantiated by the treatment’s efficacy on some other secondary end-point, in particular on additional functional scales such as ADCS-ADL and other cognitive outcomes (ACE-R, SAND). However, no significant effects were observed on behavioural disturbances, emphasizing a potential specific role for co-ultraPEAlut in modifying the progression of cognitive and functional deficits.

### Limitations

Our study has several limitations. The current study is limited by generalizability issues due to a monocentric design, the absence of specific biomarkers hindering diagnosis and monitoring treatment response and pharmacodynamic effects, the need for longer observation periods for disease-modifying treatments, and the biological and phenotypic heterogeneity of the population enrolled, including both PPA and bvFTD patients, complicating research efforts. Another limitation of our study is its conduct during the COVID-19 pandemic and the Italian lockdown (9 March 2020 to 18 May 2020 and 13 October to 26 November 2020), which necessitated the collection of certain outcome measures through telemedicine visits. While this approach allowed us to maintain and ensure participant follow-up, it may have affected the consistency and accuracy of assessments compared with in-person evaluations. Additionally, external factors associated with the pandemic, such as heightened stress levels, isolation, changes in healthcare access and alterations to daily routines, could potentially serve as confounding factors. Finally, we decide to prioritize CDR plus NACC FTLD—SoB as primary outcome measure following the initiation of the trial, based on upcoming literature findings to use functional and global composite measures that consider the diverse manifestations of these disorders.

## Conclusion

In this Phase 2 study, we showed that co-ultraPEAlut treatment is safe and may be effective in slowing down disease progression, functional decline and language deterioration in FTD. Further multicenter Phase 2/3 studies are needed to confirm the clinical validity of this promising therapeutic approach and to better define its mechanisms of action.

## Supplementary Material

fcaf080_Supplementary_Data
