# Supplementary material for: Phase 2 study of palmitoylethanolamide combined with luteoline in frontotemporal dementia patients
Source: Brain Commun. 2025 Mar 5;7(2):fcaf080. doi: 10.1093/braincomms/fcaf080 (PMC11881584; doi:10.1093/braincomms/fcaf080)
Supplement: fcaf080_Supplementary_Data [file fcaf080_supplementary_data.docx]

**Protocol and statistical analysis plan for:** Assogna M. , Di Lorenzo F., Bonnì S., Palmitoylethanolamide Combined with Luteoline in Frontotemporal Dementia patients (PEA-FTD): A Randomized Clinical Trial

Version 2.0 of 11/29/2022

The protocol was drafted before the trial began, and was amended to Version 2.0 (2022-12-14).

**This supplement contains the following items:**

**1. STUDY PROTOCOL**

1.1 ADMINISTRATIVE INFORMATION…………………………..........page 2

1.2 SYNOPSIS………………………………………………………..........page 3

1.3 ABBREVIATIONS AND DEFINITIONS……………………….........page 4

1.4 RATIONALE……………………………………………………..........page 7

1.5 OBJECTIVES………………………………………………………….page 13

1.6 INVESTIGATIONAL PLAN………………………………………….page 15

1.7 STUDY POPULATION…………………………………………….....page 19

1.8 TREATMENT……………………………………………………........page 21

1.9 EFFICACY AND SAFETY EVALUATIONS:

OUTCOME MEASURES…………………………………………………page 23

1.10 INFORMED CONSENT, ETHICAL REVIEW AND REGULATORY CONSIDERATIONS………………………………………………………page 26

**2. STATISTICAL ANALYSIS PLAN**

2.1 SAMPLE SIZE AND STATISTICAL METHODS……………………page 28

REFERENCES……………………………………………………………..page 31

1. **STUDY PROTOCOL**

**SECTION 1.1 : ADMINISTRATIVE INFORMATION**

**Title:** Palmitoylethanolamide Combined With Luteoline in Frontotemporal Dementia Patients. A Randomized Controlled Trial (PEA-FTD)

**Trial registration number:** ClinicalTrials.gov Identifier: NCT04489017

**Sap version:** V1

**Protocol version:** V1

**SAP roles and responsibility:** Giacomo Koch (P. I.), Clarissa Ferrari (statistician).

**SECTION 1.2: SYNOPSIS**

Currently, there is no effective pharmacological treatment to slow the progression of frontotemporal dementia (FTD). It has been recently proposed that neuroinflammation is an important component of FTD pathology since the early phases of the disease. For instance, progranulin acts as a mediator of the inflammatory response and its deficiency may lead to greater microglial activation and a dysregulated inflammatory response that could cause neuron death and disease progression in FTD. Normal protective microglia mediates clearance of abnormal protein aggregates, and promotes neuroregeneration. However, activated microglia, functionally impaired by abnormal protein aggregates, secretes inflammatory mediators (i.e.interleukin-1β), and co-activates astrocytes, inducing neuronal death. In TDP-43 transgenic mice a trends towards an increase of endocannabinoids receptors (CB), were also reported. These findings support the idea that the endocannabinoid signalling system, in particular the CB2 receptor, may be a target for the development of neuroprotective therapies in TDP-43 related disease. Among the endocannabinoids, palmitoylethanolamide (PEA) has shown to exert strong anti-inflammatory, immunomodulatory and neuroprotective effects. Recently, an available form of ultra-micronized PEA combined with luteoline (PEA-LUT) has gained attention for its proven anti-inflammatory and neuroprotective properties reported in neurodegenerative conditions related to FTD such as Amyotrophic Lateral Sclerosis. In this framework, it is urgent to test the efficacy of PEA-LUT on FTD patients in order to assess if targeting neuroinflammation and CB receptors could prevent neurodegeneration and restore neuronal functioning. In the current project, we hypothesize that the administration of ultra-micronized PEA-LUT may have a clinical impact in FTD patients. In particular, PEA-LUT treatment could be able to reduce disease severity progression in FTD, with a related improvement of daily living activities of affected people. Hence we planned a phase IIa 24-week, prospective, randomized, double-blind placebo controlled study. The study was designed to evaluate the efficacy, safety, and tolerability of ultra-micronized PEA-LUT versus placebo in a sample of fifty patients with probable FTD based on the International consensus clinical diagnostic criteria.

**SECTION 1.3. ABBREVIATIONS AND DEFINITIONS**

**AD:** Alzheimer’s disease

**Aβ:** amyloid- β peptides

**ACE-R:** Addenbrooke's Cognitive Examination Revised

**AChEI:** acetylcholinesterase inhibitor

**ADCS-ADL:** Alzheimer’s Disease Cooperative Study–Activities of Daily Living Inventory

**AE:** adverse event: Any untoward medical occurrence in a patient or clinical investigation subject administered a pharmaceutical product that does not necessarily have a causal relationship with this treatment. An adverse event can therefore be any unfavorable and unintended sign (including an abnormal laboratory finding, symptom, or disease temporally associated with the use of a medicinal (investigational) product, whether or not related to the medicinal (investigational) product.

**ALS:** amyotrophic lateral sclerosis

**ANOVA:** analysis of variance

**Blinding:** A procedure in which one or more parties to the trial are kept unaware of the treatment assignment(s). Blinding will remain in effect until final database lock. A double-blind study is one in which neither the patient nor any of the investigator who are involved in the treatment or clinical evaluation of the subjects are aware of the treatment received.

**CDR:** Clinical Dementia Rating Scale

**FTLD-CDR SoB:** FTLD-modified Clinical Dementia Rating scale Sum of Boxes

**CRF:** Case report form, a printed or electronic form for recording study patients’ data during a clinical study, as required by the protocol.

**CSF:** cerebrospinal fluid

**DLPFC:** dorsolateral prefrontal cortex

**Efficacy:** Efficacy is the ability of a treatment to achieve a beneficial intended result under controlled conditions.

**End of study (trial):** The date of the last visit or last scheduled procedure shown in the Study. Schedule for the last active patient in the study.

**FAB:** Frontal assessment battery

**FBI:** Frontal Behavioural Inventory

**FTD:** Frontotemporal dementia

**GABA:** Gamma-Aminobutyric acid

**GLMM:** Generalized Linear Mixed Model

**ICF**: short interval intracortical facilitation

**Informed consent:** A process by which a patient voluntarily confirms his or her willingness to participate in a particular trial, after having been informed of all aspects of the trial that are relevant to the patient’s decision to participate. Informed consent is documented by means of a written, signed, and dated informed consent form.

**Intent-to-Treat (ITT):** The principle that asserts that the effect of a treatment policy can be best assessed by evaluating on the basis of the intention to treat a patient (that is, the planned treatment regimen) rather than the actual treatment given. It has the consequence that patients allocated to a treatment group should be followed up, assessed, and analyzed as members of that group, irrespective of their compliance to the planned course of treatment.

**Investigational product**: A pharmaceutical form of an active ingredient or placebo being tested or used as a reference in a clinical trial.

**Investigator:** A person responsible for the conduct of the clinical study at a study site. If a study is conducted by a team of individuals at a study site, the investigator is the responsible leader of the team and may be called the principal investigator

**IRB/ERB:** Institutional Review Board/Ethical Review Board: a board or committee (institutional, regional, or national) composed of medical professional and nonmedical members whose responsibility is to verify that the safety, welfare, and human rights of the patients participating in a clinical study are protected.

**LICI**: long interval intracortical inhibition

**LvPPA:** logopenic variant

**MMSE:** Mini-Mental State Examination

**NfvPPA:** nonfluent/agrammatic variant

**NPI:** Neuropsychiatric Inventory

**Patient**: A study participant who has the disease or condition for which the investigational product is targeted.

**PEA-LUT:** Palmythoilethanolamide combined with luteoline

**PPA:** Primary Progressive Aphasia

**SAI:** short afferent inhibition

**SAND:** Screening for Aphasia in Neurodegeneration

**SAP:** statistical analysis plan

**SvPPA:** semantic variant

**TMS:** transcranial magnetic stimulation

**TBS:** theta-burst stimulation

**Study Entry Terms**:

**1) Screen:** The act of determining if an individual meets minimum requirements to become part of a pool of potential candidates for participation in a clinical study. In this study, screening involves medical and neurological tests, including magnetic resonance imaging or computed tomography, and lumbar puncture for CSF biomarkers analysis for diagnostic purposes.

**2) Enroll:** The act of assigning a patient to a treatment. Patients who are enrolled in the study are those who have been assigned to a treatment.

**3) Enter:** The act of obtaining informed consent for participation in a clinical study from patients deemed eligible or potentially eligible to participate in the clinical study. Patients entered into a study are those who sign the informed consent form directly or through their legally acceptable representatives.

**SECTION 1.4: STUDY RATIONALE**

Frontotemporal dementia (FTD) is a devastating neurodegenerative disorder, primarily affecting the frontal and/or temporal lobes of the brain. It is the second most frequent cause of presenile neurodegenerative dementia in those less than 65 years of age. The core FTD spectrum disorders include behavioral variant FTD (bv-FTD) characterized by prominent changes in behavior, personality and executive control (*Rascovsky et al., 2011*) and Primary Progressive Aphasia (PPA), with its clinical variants characterized by specific linguistic/cognitive deficits, i.e. nonfluent/agrammatic variant (nfvPPA), semantic variant (svPPA), and logopenic variant (lvPPA) (*Gorno-Tempini et al., 2011*). The clinical diagnosis of FTD is a challenge for clinicians since is often misdiagnosed for Alzheimer’s disease (AD) or psychiatric disorder. From symptom onset, it takes 3-4 years to reach a correct diagnosis. Abnormalities in structural imaging may be very subtle at early stages, while functional imaging changes may not be specific enough to differentiate FTD from AD at individual patient level. Currently, there is no effective pharmacological treatment to slow down the progression of FTD. Prognosis is therefore poor, and dependency on caregivers and eventual death typically occurs in a few years after diagnosis. Experimental models have highlighted the potential neurotoxic properties of tau aggregation in FTD, though recent findings have led to a renewed interest and support for an active role of inflammation in neurodegenerative processes. It has been recently proposed that neuroinflammation could be involved in specific forms of FTD since the early phases of the disease (*Heneka et al., 2014*). Thus, pathological protein aggregation and neuroinflammatory responses may well begin before patients start experiencing FTD symptoms. An increased prevalence of related immune disease has been reported in FTD patients with semantic variant primary progressive aphasia (svPPA), that were progranulin gene (GRN) mutation carriers responsible for tau-negative pathology in FTD. Progranulin acts as a mediator of the inflammatory response (*Pickford et al., 2011*), and its deficiency may lead to greater microglial activation and a dysregulated inflammatory response that could cause neuron death and disease progression in FTD. Furthermore, some biomarkers of inflammation, such as elevated cytokines e.g., tumor necrosis factor (TNF-α) in the cerebrospinal fluid (CSF), have been observed in FTD patients. Moreover, in TDP-43 transgenic mice a trend towards an increase of endocannabinoids receptors (CB) was correlated with a small reduction of Fatty Acid Amide Hydrolase (FAAH). These findings support the idea that the endocannabinoid signaling system, in particular the CB2 receptor, may serve for the development of a neuroprotective therapy in TDP-43-related disorders. Moreover, a role for microglia has also been recently debated in FTD. Normal protective microglia mediate clearance of abnormal protein (such as Aβ or tau) aggregates, remove cell debris, and promote neuroregeneration. However, activated microglia secrete inflammatory mediators (e.g., interleukin IL-1β), and co-activate astrocytes, inducing neuronal death, which further increases brain tissue damage with amplified microglial activation. Genes related to microglial activation (e.g., a variant of the triggering receptor expressed on myeloid cells 2 -TREM2) have now been associated with FTD (*Guerreiro et al., 2013*). New hypothesis has suggested that microglia could be functionally impaired by abnormal protein aggregates, leading to reduced microglial motility and phagocytic activity in vivo (*Prinz et al., 2014*). On the other hand, microglia-driven neuroinflammation could lead to the formation of tau aggregation. In FTD, the neuronal and axonal degeneration are sufficient to induce microglial activation. All these findings support the idea that neuroinflammation has a detrimental role in FTD and that novel drugs targeting neuroinflammation, could potentially be useful in FTD.

Palmitoylethanolamide (PEA) is a saturated N–acylethanolamide belonging to the family of endocannabinoids. Broadly speaking, endocannabinoids are endogenous ligands binding to cannabinoid receptors and thereby engaging in respective neuromodulatory activities *(Di Marzo et al., 1998).* PEA, in particular, has been reported to possess strong anti-inflammatory *(Solorzano et al., 2009; Lo Verme et al., 2005)* properties. More precisely, ischemic injury models attribute a potential role to PEA in protecting neural cells against glutamate toxicity. The ability of PEA to modulate neuroinflammatory responses has been applied to several pathological conditions including pain *(Hesselink and Hekker, 2012; Indraccolo and Barbieri, 2010),* spinal cord injury (*Genovese et al., 2008*) and amyotrophic lateral sclerosis (ALS) (*Palma et al., 2016*). Notably, neither serious adverse effects nor drug interactions are observed with this natural compound. Animal models of neurodegeneration recently demonstrated PEA anti-inflammatory and neuroprotective properties, as well as its ability to preserve memory function in rodent models of AD *(Scuderi et al., 2014).* The central role of PEA in mediating neuroinflammation is thought to depend partially on its powerful action on microglia cells. Microglia are activated in response to a number of different pathological states within the CNS including neurodegenerative disorders such as Alzheimer's disease, Parkinson's disease, multiple sclerosis, ALS. Activated microglia elaborates a potentially lethal cocktail of compounds capable of damaging neurons, oligodendrocytes or extracellular matrix molecules, and depletion or blockade of microglia and macrophages prevents disease progression *(Kim et al., 2005).* Microglia/macrophages, however, may deliver trophic factors, and support myelin regeneration by phagocytic removal of obstructive myelin debris or through activation and recruitment of endogenous oligodendrocyte precursor cells to the lesion site *(Kotter et al., 2005).*

As suggested in a number of studies, PEA may be a ligand for peroxisome proliferator activated receptor α (PPARα), one of a group of nuclear receptor proteins that function as transcription factors regulating the expression of genes. The α‐ and γ‐isoforms of PPAR in particular are associated with pro‐inflammatory events. An ‘entourage effect’ has been hypothesized to explain the pharmacological actions of PEA, whereby PEA enhances the anti‐inflammatory and anti‐nociceptive activity of other endogenous compounds by potentiating their affinity for a receptor or by inhibiting their metabolic degradation. In particular, anandamide is a candidate molecule, as it possesses anti‐inflammatory and anti‐nociceptive effects *(Skaper et al., 2014).* More recently, it was found that ultra-micronized PEA combined with luteoline (PEA-LUT) enhances GABA transmission and triggers a parallel synthesis of 2-AG at the postsynaptic site, that in turn acts in a retrograde manner to inhibit GABA release through the stimulation of presynaptic CB1Rs. This electrophysiological study identifies a previously unrecognized function of PEA, demonstrating that GABAergic transmission is under the control of this compound and revealing that PEA modulates the release of the endocannabinoid 2-AG.

Overall, PEA fulfils the criteria for a favorable candidate as an adjunctive therapeutic agent for neurodegenerative disorders such as FTD, potentially controlling neuroinflammation and GABAergic neurotransmission. Currently, GABAergic neurotransmission is evaluable in-vivo by means of well-established neurophysiological protocols that adopt transcranial magnetic stimulation (TMS). Starting from the evidence that an impaired GABA neurotransmission is implicated in FTD, as demonstrated by the toxic effects mediated by tau and TDP-43 on GABAergic interneurons, recent TMS studies have been focused on the evaluation of GABAergic neurotransmission in FTD patients *(Benussi et al., 2017).* Additionally, GABAergic inhibitory neurons result to play a key role in the regulation of cortical oscillatory dynamics *(Fries, 2009; Bastos et al., 2012),* including the generation of gamma oscillations and regulation of the magnitude and frequency of these oscillations *(Buzsaki and Wang, 2012).* At this regard, several evidence highlight how GABA activity is essential for coordinating information transfer and information processing in the brain and the restoring of synaptic GABA levels increases the gamma oscillatory power in dorsolateral prefrontal cortex (DLPFC) *(Kujala et al., 2015)* with paralleled clinical improvements. Thus, there is a well-established evidence that gamma oscillations are reduced in the frontal lobes of FTD patients and that changes in cross-frequency coupling between regions of the frontal cortical network are related to behavioural control *(Hughes et al., 2018)*.

Based on this premises we recently performed a pilot study to investigate the efficacy and safety of PEA-LUT at the oral dosage of 700 mg x 2/day in a sample of 18 newly diagnosed FTD patients evaluated with clinical and behavioral\cognitive scales at baseline and after one month of treatment PEA-LUT. Patients were evaluated at baseline and after one month of treatment with PEA-LUT with the following scales: NPI, MMSE, FAB, ADL/IADL, FTLD-CDR SoB. We observed a significant amelioration in the behavioral symptoms as measured by changes from baseline in the NPI mean score (pre:15.87±4.01 vs post: 8.50±1.99; p=0.018). Moreover, we found surprisingly an improvement in frontal executive functions as revealed by an improvement in the FAB score (pre: 6.4±1.54 vs post: 7.95±1.56; p=0.038). There was also a trend for amelioration in the MMSE (pre: 18.6±3.34 vs post: 19.6±3.33; p=0.23). To non-invasively assess intracortical circuits of the primary motor cortex (M1), we used paired-pulse transcranial magnetic stimulation (ppTMS) (*Kujirai et al., 1993; Ziemann et al., 1996*). Specifically, we investigated short interval intracortical inhibition (SICI) to investigate GABA(A)-ergic neurotrasmission; short interval intracortical facilitation (ICF), to investigate glutamatergic neurotransmission and long interval intracortical inhibition (LICI), to investigate GABA(B)-ergic neurotransmission. We also investigated short latency afferent inhibition (SAI), which reflects cholinergic neurotransmission (*Tokimura et al., 2000*). Finally, to probe plasticity mechanisms, we used a novel form of TMS, namely theta-burst stimulation (TBS), to induce long-term potentiation (LTP) of M1. These measures have been evaluated by means of motor-evoked potentials (MEPs), a well-known index of corticospinal excitability (*Barker et al., 1985*).

To evaluate cortical activity, in terms of TMS-evoked potentials (TEPs) and oscillations in the left and right dorsolateral prefrontal cortex (DLPFC) we used TMS-EEG. Indeed, it has been suggested that early TEPs originate from GABA(A)-mediated and GABA(B)-mediated inhibitory post synaptic potentials (IPSPs) (*Rogasch et al., 2013*). On the other hand, TMS-evoked oscillations allow to investigate the natural frequency of a target area and to assess the thalamo-cortical circuits. After one month of PEA-LUT treatment, we observed a significant amelioration in the behavioural symptoms and in frontal executive functions. Neurophysiological results showed a remarkable improvement of GABA(B) activity as revealed by a restoration of decreased LICI. We also observed an increase of LTP- like cortical plasticity after PEA-LUT treatment. These results were quite specific since we did not find any change in the other protocols assessing GABA(A)-ergic activity (SICI) nor cholinergic activity (SAI). We also found a significant increase in TMS-evoked oscillations in both left and right DLPFC. In particular, such increase was prominent for high-frequency oscillations, such as beta (14-30 Hz) and gamma power (30-45 Hz). TEPs evaluation revealed a significant increase of cortical excitability both in left and right DLPFC, in particular in GABA(B)-mediated TEPs, between 60 and 100 ms after TMS. Taken together, these preliminary results seems to suggest that PEA-LUT could be able to reduce behavioural disturbances and improve executive functions; moreover these results show that one month of PEA-LUT can restore cortical plasticity and GABA(B) activity, which seem to be compromised in FTD patients (*Murley et al., 2018*). Finally PEA-LUT is able to restore high-frequency oscillations that are reduced between the frontal lobes of FTD patients.

In the current project, we hypothesize that the administration of ultra-micronized PEA-LUT may have a clinical impact in FTD patients. In particular, PEA-LUT treatment could be able to reduce disease severity progression in FTD, with a related improvement of daily living activities of affected people. Potentially the proposed project could provide a valid treatment for cognitive and behavioural dysfunction in FTD patients, with consistent impact for the National Health Systems and minimum cost for the patients. Moreover, the application of recent neurophysiological tools, such as the combined use of transcranial magnetic stimulation (TMS) during electroencephalography (EEG) will allow us to understand how the administration of PEA-LUT may modulate the cortical activity of the dorsolateral prefrontal cortex in FTD patients, defining the neurophysiological biomarkers of clinical improvement.

**SECTION 1.5: OBJECTIVES**

The study is designed to evaluate the efficacy and safety of Co-ultramicronized PEA-LUT administration at the oral dosage of 700 mg x 2/day for 24 weeks in newly diagnosed FTD patients. Fifty consecutive patients (40-85 years) will be recruited and will be randomized to PEA-LUT or placebo administration. To evaluate the cognitive effects of PEA-LUT we will use a battery of cognitive tests assessing global cognition, executive functions and behavior that will be performed at Baseline before starting treatment and repeated on-treatment at weeks 4, 12 and 24.

**Primary objective:**

The primary objective of this study is to test the hypothesis that therapy with PEA-LUT could have a relevant clinical impact on global disease severity in FTD patients as compared with placebo. The primary objective will be evaluated using a study endpoint at 24 weeks after initiation of treatment. The primary objective will be assessed using a Generalized Linear Mixed Model (GLMM) for repeated measures on the primary outcome: FTLD-modified Clinical Dementia Rating scale Sum of Boxes (FTLD-CDR SoB), in which the specific hypothesis is that the global disease severity progression at the end of the treatment phase for PEA-LUT will be significantly less than that for placebo.

**Secondary Objective:**

The secondary objectives of this study are as follows:

- To test the hypothesis that PEA-LUT will slow the rate of decline associated with FTD in the activities of daily living, as assessed with the Alzheimer's disease Cooperative Study Activities of Daily Living (ADCS-ADL), using a GLMM for repeated measures.
- To assess benefit of treatment with PEA-LUT on frontal cognitive functions, as demonstrated through the Frontal assessment battery (FAB), using a GLMM for repeated measures.
- To evaluate efficacy of treatment with PEA-LUT on behavior, as demonstrated through the Neuropsychiatric Inventory (NPI) and Frontal Behavioral Inventory (FBI), using a GLMM for repeated measures.
- To evaluate efficacy of treatment with PEA-LUT on language function, as demonstrated through the Screening for Aphasia in Neurodegeneration (SAND) using a GLMM for repeated measures.
- To evaluate efficacy of treatment with PEA-LUT on global cognition, as demonstrated through the Addenbrooke's Cognitive Examination Revised (ACE-R) and Mini Mental State Examination (MMSE) using a GLMM for repeated measures.
- To evaluate changes from Baseline to Week 24 in the TMS/EEG protocol, to evaluate effects of PEA-LUT on DLPFC cortical activity, oscillatory activity and connectivity.
- Nature, frequency and severity of adverse events (AEs): to assess the safety and tolerability of PEA-LUT given for up to 24 Weeks.

**SECTION 1.6: INVESTIGATIONAL PLAN**

**Summary of study design**

This is a 24-week, randomized, double-blind, placebo-controlled, Phase 2 study, comparing co-ultramicronized PEA-LUT (GLIALIA, EPITECH group) with placebo for 24 weeks in approximately 50 patients with probable FTD.

Patients will be assigned randomized in a 1:1 ratio to receive Palmythoilethanolamide combined with Lutheoline oral suspension at the dosage of 700 mg+ 70 mg twice every day or placebo for 24 weeks. Participants will be instructed to take two sachets of oral suspension per day (one in the morning and one in the evening). The primary hypothesis being tested is that PEA-LUT will slow global disease progression, the cognitive and functional decline of FTD as compared with placebo.

**Timing of outcomes assessment**

- Preliminary screening
- Review of inclusion and exclusion criteria
- Informed consent obtained
- Demography
- Family and medical history
- Prior and concomitant medications
- Randomization and group assignment

**Baseline evaluation (Day 1)**

- Clinical assessment
- Neurophysiological assessment

**Post-treatment evaluation (Day 168-week 24)**

- Clinical assessment
- Neurophysiological assessment

**Screening Phase**

At or before Visit 1, the study will be explained to the patient and caregiver. In the screening phase, all patients will undergo an extensive clinical investigation, including interviews on their medical history, a full neurological examination, the CDR-FTD, a complete blood screening, neuropsychological assessment, neuropsychiatric evaluation, and magnetic resonance imaging. In the 30 days before entering the study, subjects will have to be clear of treatment with any drug inducing modulatory effects on the cerebral cortex excitability, such as antidepressants, benzodiazepines, anti-epileptic drugs, or neuroleptics. All the patients will perform lumbar puncture for CSF biomarkers analysis for diagnostic purposes and to rule out AD pathology (Dubois et al., 2014) and a [18F] fluorodeoxyglucose (FDG) PET scan to evidence atrophy/hypometabolism.

All participants will sign a written informed consent after receiving an extensive disclosure of the experimental details.

**Evaluation Phase**

During the week before starting PEA-LUT administration, and during the week at the end of 4th (W4), 12th week (W12) and 24th week (W24) FTD patients will undergo clinical assessment. Neurophysiological assessment will be performed at baseline and W24 and the evaluation will be done in two different days.

1) Clinical Assessment: before and after the 24 weeks of treatment FTLD-CDR SoB, FAB, MMSE, SAND, ACE-R, FBI, ADCS-ADL, FAB and NPI will be administered.

2) Neurophysiological assessment: for TMS-EEG recordings, a TMS-compatible EEG equipment will be used for recording EEG activity from the scalp (BrainAmp, BrainProducts). The EEG will be continuously acquired from 61 scalp sites positioned according to the 10-20 International System. TMS-EEG recordings will be performed over the right and left DLPFC. To precisely position the coil over the cortical sites across different sessions, a neuronavigation system (Softaxic, E.M.S.) will be used. During the TMS-EEG session, DLPFC will be stimulated with 80 single TMS pulses (Koch et al., 2018).

**Treatment Phase**

After recruitment and baseline assessments, FTD patients will be assigned to PEA-LUT or PLC. We will use the Glialia® 700 m+70 mg oral sachets administered twice every day. The first mechanism of action for PEA was proposed by Rita Levi‐Montalcini's research group, who suggested that PEA acts via ‘Autacoid Local Injury Antagonism (ALIA)’ to down‐regulate mast cell activation (*Levi‐Montalcini et al., 1996*). Later, the existence of a ‘direct receptor‐mediated mechanism’ was proposed, and several studies demonstrated that PEA can act via direct activation of at least two different receptors: the PPAR‐α (*Lo Verme et al., 2005a*) and the orphan GPCR 55 (GPR55) (*Ryberg et al., 2007*). It was originally thought that PEA could also be a CB2 receptor agonist (*Facci et al., 1995*), but subsequent studies revealed that PEA has only very weak affinity for this receptor, explaining why some of its anti‐inflammatory effects are not blocked by CB2 receptor antagonists (*Costa et al., 2002*). As a result, the theory of the ‘entourage’ effect was put forward to raise the possibility that PEA could produce indirect receptor‐mediated effects (De Petrocellis et al., 2001; Di Marzo et al., 2001; Ho et al., 2008). For example, PEA, through the inhibition of the expression of FAAH, the enzyme responsible for the degradation of the endogenous cannabinoid receptor ligand (or endocannabinoid), anandamide (AEA) (*Di Marzo et al., 2001*), may indirectly activate CB2 and CB1 receptors (*Di Marzo et al., 2001; Petrosino et al., 2016a*). Likewise, PEA can indirectly activate the transient receptor potential vanilloid receptor type 1 (TRPV1) channels, which are also targets for the endocannabinoids (*Zygmunt et al., 1999, 2013*). In addition, PEA is also able to increase AEA‐ or 2‐AG‐induced TRPV1 activation and desensitization (*De Petrocellis et al., 2001; Di Marzo et al., 2001*; *Petrosino et al., 2016a*). More recently, it has also been demonstrated that PEA can activate TRPV1 channels or increase the expression of CB2 receptors via PPAR‐α receptors (*Ambrosino et al., 2013; Ambrosino et al., 2014*). In summary, these results suggest that PEA does not operate through just one main mechanism of action. Instead, synergistic interactions among several mechanisms often seem necessary so that PEA can produce its important therapeutic effects, acting on neuroinflammation, both in the central and the peripheral nervous system. All treatments will be administered for 24 weeks with no interruptions. The placebo sachets packaging will be identical to the PEA-LUT sachets.

**SECTION 1.7: STUDY POPULATION**

Eligible patients will be males and females with probable Frontotemporal Dementia according to current diagnostic criteria (Rascovsky et al., 2011, Gorno-Tempini et al. 2011) as specified in the entry criteria that follow. Entered patients who meet all of the inclusion criteria and are not excluded by any of the exclusion criteria will be randomized and proceed to Evaluation Phase.

**Inclusion Criteria**

Patients are eligible to be included in the study only if they meet all of the following criteria:

1. The patient has a diagnosis of probable Frontotemporal dementia based on the International consensus clinical diagnostic criteria described by Rascovsky et al., 2011, Gorno Tempini et al.2011.
2. The patient is a man or a woman, aged from 40 to 85 years.
3. The patient has a Clinical Dementia Rating-FTD (CDR-FTD) total score of ≤2 at Screening.
4. The patient has not been treated with acetylcholinesterase inhibitor (AChEI), i.e., donepezil, galantamine, or rivastigmine, at the time of screening.
5. The patient is able to comply with the study procedures in the view of the investigator.
6. Evidence of frontotemporal hypometabolism at PET imaging.
7. Evidence of amyloid markers excluding Alzheimer's disease (cerebrospinal fluid Abeta/Tau dosages or amyloid PET imaging).
8. The patient (or if applicable the legally acceptable representative if different from the responsible caregiver) and the responsible caregiver have signed the Informed Consent Form.
9. Has at least one identified adult caregiver who is able to provide meaningful assessment of changes in subject behavior and function over time and provide information on safety and tolerability, and is able to verify daily compliance with study drug

**Exclusion Criteria:**

1. Significant neurodegenerative disorder of the central nervous system other than FTD e.g., Alzheimer's disease, Lewy body dementia, Parkinson's disease, multiple sclerosis, progressive supranuclear palsy, normal pressure hydrocephalus, Huntington's disease, any condition directly or indirectly caused by Transmissible Spongiform Encephalopathy (TSE), Creutzfeldt-Jakob Disease (CJD), variant Creutzfeldt-Jakob Disease (vCJD), or new variant Creutzfeldt-Jakob Disease (nvCJD)
2. Significant intracranial focal or vascular pathology seen on brain MRI scan within a maximum of 6 months before Baseline leading to a diagnosis other than probable FTD.
3. The patients has history of seizure (with the exception of febrile seizures in childhood).
4. Metal implants in the head (except dental), pacemaker, cochlear implants, or any other non-removable items that are contraindications to MR imaging.
5. Treatment currently or within 3 months before Baseline with any of the following medications: Typical and Atypical antipsychotics (i.e., Clozapine, Olanzapine); Antiepileptics drugs (i.e., Carbamazepine, Primidone, Pregabalin, Gabapentin); Antidepressants (i.e., Citalopram, Duolxetine, Paroxetine).

**Screening Procedures**

Eligibility of participants in the study will be based on the results of sequential screening procedures including medical history, physical examination, and neurological evaluation including MRI or PET scan. The screening period allows patients adequate time to decide whether they wish to participate in the study

**SECTION 1.8: TREATMENT**

**1.8.1. Treatments administrated**

Treatment is given to all patients for 24 weeks with no interruption.

The investigational drug o-ultramicronized PEA-LUT (Glialia) is provided as oral sachets.

Placebo is identical in appearance and will be contained in identical packages

At each post-Baseline visit during the treatment period, the number of sachets dispensed to the patient/caregiver is recorded. Patient compliance with prescribed study drug is also assessed at each visit by questioning the patient and caregiver. Compliance data, including dates of any dose deviations and/or interruptions, and any other pertinent information are recorded in the source documentation.

Use of the following treatments are not allowed in combination with study treatment, due to confounding of efficacy and/or potential interaction with study treatment:

- Any investigational drug: disallowed <60gg from screening

- General anesthetics are disallowed during the study except in case of emergency procedures requiring anesthesia. Episodic use of local anesthetics is allowed.

- Antidepressant: stable treatment with no modification of dose with selective serotonin reuptake inhibitors (SSRIs), venlafaxine, moclobemide and mirtazapine for at least 3 months prior to the Screening Visit acceptable. Paroxetine and duloxetine should be used with caution. Dose modifications and initiation of treatment not allowed during the study.

- Antiparkinsonian agents (e.g., levodopa, dopamine agonists, COMT inhibitors, amantadine, monoamine oxidase B inhibitors, anticholinergics etc.): disallowed for 6 months prior to screening and during the study.

- Antipsychotics typical and atypical: disallowed for 3 months prior to screening and during the study.

- Sedative/hypnotics: disallowed for 3 months prior to screening and during the study. Melatonin will be allowed anytime.

**SECTION 1.9. EFFICACY AND SAFETY EVALUATIONS: OUTCOME MEASURES**

**Primary Efficacy Measure**

**1)** Global disease severity [Time Frame: change from baseline to Week 24]: Clinical Dementia Rating Scale- Frontotemporal dementia Sum of Boxes. The scores range from 0-24 with a higher score meaning higher disease severity. A modification in the pre-specified primary endpoint was done in this version based on upcoming literature findings (*Staffaroni et al., 2019; Desmarais et al 2019*). Indeed, emerging evidence suggests that, for trials potentially involving patients with any of the FTD syndromes, priority should be given to functional and global composite measures. These measures take into account the diverse manifestations of these disorders and are consider more attractive clinical outcome metrics due to their ability to yield comparable sample size estimates across all groups and their inherent clinical significance (Staffaroni et al., 2019; Desmarais et al., 2019). On the contrary, NPI should not be regarded as a reliable measure in this syndrome (Knopman et al., 2008; Staffaroni et al., 2019), possibly due to the inter-subject variability of behavioral phenotypes (Ranasinghe et al., 2016). The decision to prioritize CDR plus NACC FTLD - SoB was made by the Principal Investigator (G.K) independently of trial data and prior to unblinding.

**Secondary Efficacy Measures**

**1)** Activities of daily living [Time Frame: change from baseline to Week 24]: AD Cooperative Study - Activities of Daily Living (ADCS-ADL). The ADCS-ADL includes 23 items that were derived from a larger set of items describing performance of activities of daily living (ADL) by AD patients (*Galasko et al., 1997*). The ADCS contained 23 items covering physical and mental functioning and independence in self-care. For each basic ADL (eating, walking, toileting, bathing, grooming, selecting, clothes), there is a forced choice of best response. All other ADL consist of a main question followed by subquestions (descriptors). The scores range from 0 to 78, with lower values indicating greater disability.

**2)** Neuropsychiatric evaluation [Time Frame: change from baseline to Week 24]: Neuropsychiatric Inventory (NPI). The NPI assesses behavioral disturbances in dementia. The NPI is reliable and valid (*Cummings, 1994*) and has been reported to be sensitive to the effects of tacrine (*Kaufer et al., 1996*). The NPI measures the following behavioral areas: delusions, hallucinations, agitation, depression, anxiety, euphoria, apathy, disinhibition, irritability, aberrant motor behavior, night time behaviors, and eating disorders. Symptoms are rated in terms of frequency (0-4) and severity (0-3). Total score range is 0-144, where higher scores indicate higher behavioral psychopathology.

**3)** Neuropsychiatric evaluation [Time Frame: change from baseline to Week 24]: Frontal Behavioural Inventory (FBI). The scores range from 0-72 with a higher score meaning more severe behavioural disturbances.

**4)** Frontal cognitive functions [Time Frame: change from baseline to Week 24]: Frontal assessment battery (FAB). The Frontal Assessment Battery is a brief screening tool for the assessment of frontal lobe function *(Appollonio et al., 2005*). It is used to measure abstraction, fluency, impulsivity and primitive reflexes in patients who have a suspected frontal lobe deficit. A higher score indicates better performance. The FAB has been used to assess the effects of rivastigmine on frontal functions in AD patients (*Park et al., 2017*).

**5)** Language function evaluation [Time Frame: 24 weeks ] Screening for aphasia in Neurodegeneration (SAND) scale (*Catricalà et al., 2017*) . The scores range from 0-84 with a higher score meaning less severe language deficits.

**6)** Global Cognition [Time Frame: change from baseline to week 24]: Addenbrooke's Cognitive Examination Revised (ACE-R)The scores range from 0-100 with a higher score meaning less cognitive impairment.

**7)** Global Cognition [Time Frame: change from baseline to week 24]: Mini Mental State Examination (MMSE). The scores range from 0-30 with a higher score meaning less cognitive impairment.

**8)** TMS-evoked EEG responses, in terms of potentials (i.e., TEPs) and oscillations (i.e., TRSP) as a novel probe of cortical excitability changes induced by PEA-LUT. TEPs and TRSP will be computed by stimulating the left and right DLPFC and simultaneous electroencephalography recordings over all the scalp.

**1.9.1 SAFETY EVALUATIONS**

Investigators are responsible for monitoring the safety of patients who have entered this study. The investigator is responsible for the appropriate medical care of patients during the study. The investigator remains responsible for following, through an appropriate health care option, AEs that are serious or that caused the patient to discontinue before completing the study. The patient should be followed until the event is resolved or explained. Frequency of follow-up evaluation is left to the discretion of the investigator.

**1.9.2.1 Adverse Events and Other Safety Measures**

A clinical study AE is any untoward medical event associated with the use of a drug or drug delivery system in humans. AE collection begins after the patient has signed informed consent and has received study drug. If a patient experiences an AE after signing informed consent, but before receiving study drug, the event will not be collected unless the investigator feels the event may have been caused by a protocol procedure.

At each clinic visit, AEs will be recorded, vital signs will be measured and physical and neurological examination will be performed. An independent Data Monitoring Committee will monitor the patients' safety according to the Data Monitoring Committee Charter.

**SECTION 1.10. INFORMED CONSENT, ETHICAL REVIEW AND REGULATORY CONSIDERATIONS**

**1.10.1 Informed Consent**

The principal investigator is responsible for ensuring that the patient understands the potential risks and benefits of participating in the study, including answering any questions the patient may have throughout the study and sharing in a timely manner any new information that may be relevant to the patient’s willingness to continue his or her participation in the trial. The Informed Consent Form (ICF) will be used to explain the potential risks and benefits of study participation to the patient/caregivers in simple terms before the patient is entered into the study, and to document that the patient is satisfied with his or her understanding of the risks and benefits of participating in the study and desires to participate in the study. The investigator is responsible for ensuring that informed consent is given by each patient or legal representative. This includes obtaining the appropriate signatures and dates on the ICF prior to the performance of any protocol procedures and prior to the administration of investigational product.

**1.10.2 Ethical Review and Regulatory Considerations**

This study will be conducted in accordance with:

1) consensus ethics principles derived from international ethics guidelines, including the Declaration of Helsinki and Council for International Organizations of Medical Sciences (CIOMS) International Ethical Guidelines

2) Institutional Review Board (IRB) Guidelines

3) Applicable laws and regulations.

The investigator or designee will promptly submit the protocol to applicable ERB. An identification code assigned by the investigator to each patient will be used in lieu of the patient’s name to protect the patient’s identity when reporting AEs and/or other trial-related data.

**1.10.3 Investigator Information**

Physicians with expertise in neurology or geriatrics who have clearly documented extensive experience in AD and FTD trials will participate as investigators in this clinical study. In addition, licensed clinicians (neuropsychologists) who have clearly documented extensive experience in AD and FTD trials may participate as investigators in this clinical study. Cognitive assessments must be administered by an individual trained in the use of these instruments. Neurophysiological recordings and analysis must be performed by experts in this field. All lumbar punctures must be performed by an appropriately trained individual.

**2. STATISTICAL ANALYSIS PLAN**

**2.1. Determination of Sample Size**

The appropriateness of our sample size, i.e. 48 randomly assigned patients (25 to PEA- LUT treatment group and 23 to placebo group), was based on the effect size obtained in a preliminary study by our group using a shorter version of our protocol. Considering the cognitive and behavioural results of this study, we obtained a medium effect size of 0.93 (computed as the difference between the post-treatment and the pre-treatment mean over the pooled standard deviation). With these effect sizes, adopting a two-tailed paired Wilcoxon signed-rank test, with type I error alpha=0.05, the minimum sample for reaching a power (1-β) of 0.95 was estimated equal to n=18 patients per group. We decided to increase our sample size at least of 30% considering possible drop-outs.

**2.2. Method of Assignment to Treatment**

Subjects who meet all criteria for enrollment will be randomized to double-blind treatment. Randomization will be performed and assigned independently by a statistician working in an independent institution, held centrally, and not divulged to any other person involved in the trial until after database lock. We will adopt a simple randomization procedure scheme.

**2.3 Statistical and Analytical Plans**

**2.3.1. General Considerations**

Unless otherwise noted, all tests of treatment effects will be conducted at a 2-sided alpha level of 0.05; 2-sided confidence intervals will be displayed with a 95% confidence level. All tests of interactions between treatment and other factors will be conducted at an alpha level of 0.05. All analyses will follow the intent-to-treat (ITT) principle unless otherwise specified. An ITT analysis is an analysis of data by the groups to which subjects are assigned by random allocation, even if the subject does not take the assigned treatment, does not receive the correct treatment, or otherwise does not follow the protocol.

**2.3.2. Analysis Populations**

Baseline characteristics as well as the co-primary and secondary efficacy measures will be summarized for the ITT and per-protocol populations by treatment group and overall. Tabulations of the number and percentage of subjects included in each analysis set, by treatment group and overall, will be provided. Reasons for exclusion from analysis datasets will also be provided.

**2.3.3. Patient Characteristics**

The patient’s age, gender, height, body weight, tobacco use, alcohol use, caffeine use, years of education, work status, time since onset of first FTD symptoms, time since diagnosis, MMSE and CDR at Visit 1, and concomitant drug use at baseline will be recorded. Baseline characteristics will be summarized for the ITT and per-protocol populations by treatment group and overall. Summaries will include descriptive statistics for continuous and categorical measures. Pearson’s chi-square test will be used for treatment group comparisons of categorical data. For continuous data, t-test or corresponding non-parametric test (Mann-Whitney) will be used for the group comparing.

**2.3.4. Analysis of Efficacy Outcomes**

Normality assumption of end-points variables will be assessed by inspection of the distribution plots and by Kolmogorov-Smirnov and Shapiro-Wilk tests. Preliminary descriptive analyses will be performed by means, frequencies, standard deviations (SD) and percentages. Comparison of socio-demographic and clinical features between the study groups at baseline will be performed through t-test or Mann-Whitney tests. The longitudinal assessment of the end-points across groups will be performed through Generalized Linear Mixed Model (GLMM) for repeated measures with random intercept and random slope to account for individual differences at baseline as well as for individual change after the treatment. GLMMs will be applied to FTLD-CDR SoB and the other efficacy outcome measures, MMSE, ACE-R, FBI, SAND, ADCS-ADL, FAB and NPI, as dependent variables (one model for each outcome measure), and group, time and group x time interaction as independent factors. In detail, analysis of residuals (after adjustment for variables that mainly affect the outcomes: age and education) led us to adopt GLMMs for Gaussian data with identity link function for FTLD-CDR SoB, SAND, FBI, ACE-R, MMSE, ADCS-ADL and FAB; whereas GLMM for Negative binomial data, with log-link function, will be used for NPI. The GLMMs on MMSE and FAB will be adjusted for age and education.

TEPs analysis will be conducted in two steps. First, to identify the exact the TEP time windows at which there will be a significant difference, we will perform multiple t-tests (corrected with false discovery rate method) at each time point of the TEP waveform from -100 to 300 ms after TMS. This analysis will be conducted for each group (PEA-LUT group vs placebo) and for each evaluation (baseline W0 vs W24). Once detected the significant time windows, we will compare the mean TMS-evoked activity (dependent variable) within these intervals with a generalized linear mixed model (GLMM -for Gaussian data-) for repeated measures with group (real vs. sham), time (W0 vs. W24) and “window” as independent factors. TRSP analysis will be conducted with dependent t-tests comparing the different frequency bands (alpha, beta, gamma, theta) in the two time points of evaluation (W0 and W24). Safety analyses for the treatment period will include comparisons between PEA-LUT group and placebo groups by 2-sided 0.05 significance level Chi-square tests. Statistical analyses will be performed with IBM SPSS statistics, version 24.0 and R software; significance alpha level test is set up at 0.05.

**Adjustment for covariates**

Possible confounders will be chosen based on literature. The evaluation of the effect of such confounders on primary and secondary outcomes will be carried out through an ad-hoc linear and generalized linear model and a corresponding analysis of residuals.

**References**

- Ambrosino P, Soldovieri MV, De Maria M, Russo C, Taglialatela M. Functional and biochemical interaction between PPARα receptors and TRPV1 channels: Potential role in PPARα agonists-mediated analgesia. Pharmacological research. 2014 Sep 1;87:113-22.
- Ambrosino P, Soldovieri MV, Russo C, Taglialatela M. Activation and desensitization of TRPV1 channels in sensory neurons by the PPARα agonist palmitoylethanolamide. British journal of pharmacology. 2013 Mar 1;168(6):1430-44.
- Barker AT, Jalinous R, Freeston IL. Non-invasive magnetic stimulation of human motor cortex. The Lancet. 1985 May 11;325(8437):1106-7.
- Bastos AM, Usrey WM, Adams RA, Mangun GR, Fries P, Friston KJ. Canonical microcircuits for predictive coding. Neuron. 2012 Nov 21;76(4):695-711.
- Benussi A, Di Lorenzo F, Dell'Era V, Cosseddu M, Alberici A, Caratozzolo S, Cotelli MS, Micheli A, Rozzini L, Depari A, Flammini A, Ponzo V, Martorana A, Caltagirone C, Padovani A, Koch G, Borroni B. Transcranial magnetic stimulation distinguishes Alzheimer disease from frontotemporal dementia. Neurology. 2017 Aug 15;89(7):665-672.
- Buzsáki G, Wang XJ. Mechanisms of gamma oscillations. Annual review of neuroscience. 2012 Jul 21;35:203-25.
- Casula EP, Pellicciari MC, Picazio S, Caltagirone C, Koch G. Spike-timing-dependent plasticity in the human dorso-lateral prefrontal cortex. Neuroimage. 2016 Dec 1;143:204-13.
- Casula EP, Tarantino V, Basso D, Arcara G, Marino G, Toffolo GM, Rothwell JC, Bisiacchi PS. Low-frequency rTMS inhibitory effects in the primary motor cortex: Insights from TMS-evoked potentials. Neuroimage. 2014 Sep 1;98:225-32.
- Catricalà E, Gobbi E, Battista P, Miozzo A, Polito C, Boschi V, Esposito V, Cuoco S, Barone P, Sorbi S, Cappa SF. SAND: a Screening for Aphasia in NeuroDegeneration. Development and normative data. Neurological Sciences. 2017 Aug 1;38(8):1469-83.
- Costa B, Conti S, Giagnoni G, Colleoni M (2002). Therapeutic effect of the endogenous fatty acid amide, palmitoylethanolamide, in rat acute inflammation: inhibition of nitric oxide and cyclo‐oxygenase systems. Br J Pharmacol 137: 413–420.
- Cummings JL. The Neuropsychiatric Inventory Assessing psychopathology in dementia patients. Neurology. 1997 May 1;48(5 Suppl 6):10S-6S.
- Delbeuck X, Van der Linden M, Collette F. Alzheimer'disease as a disconnection syndrome? Neuropsychology review. 2003 Jun 1;13(2):79-92.
- De Petrocellis L, Davis JB, Di Marzo V (2001). Palmitoylethanolamide enhances anandamide stimulation of human vanilloid VR1 receptors. FEBS Lett 506: 253–256
- Desmarais P, Rohrer JD, Nguyen QD, Herrmann N, Stuss DT, Lang AE, Boxer AL, Dickerson BC, Rosen H, van Swieten JC, Meeter LH, Borroni B, Tartaglia MC, Feldman HH, Black SE, Masellis M. Therapeutic trial design for frontotemporal dementia and related disorders. J Neurol Neurosurg Psychiatry. 2019 Apr;90(4):412-423. doi: 10.1136/jnnp-2018-318603. Epub 2018 Oct 25. PMID: 30361298.
- Di Marzo V, Melck D, Orlando P, Bisogno T, Zagoory O, Bifulco M et al. (2001). Palmitoylethanolamide inhibits the expression of fatty acid amide hydrolase and enhances the anti‐proliferative effect of anandamide in human breast cancer cells. Biochem J 358: 249–255
- Di Marzo V, De Petrocellis L (2010). Endocannabinoids as regulators of transient receptor potential (TRP) channels: a further opportunity to develop new endocannabinoid‐based therapeutic drugs. Curr Med Chem 17: 1430–1449.
- Di Lazzaro V, Oliviero A, Profice P, Pennisi MA, Di Giovanni S, Zito G, Tonali P, Rothwell JC. Muscarinic receptor blockade has differential effects on the excitability of intracortical circuits in the human motor cortex. Experimental Brain Research. 2000 Dec 1;135(4):455-61.
- Di Lazzaro V, Rothwell JC. Corticospinal activity evoked and modulated by non‐invasive stimulation of the intact human motor cortex. The Journal of physiology. 2014 Oct 1;592(19):4115-28.
- Di Marzo V, Bisogno T, De Petrocellis L, Brandi I, Jefferson RG, Winckler RL, Davis JB, Dasse O, Mahadevan A, Razdan RK, Martin BR. Highly selective CB 1 cannabinoid receptor ligands and novel CB 1/VR 1 vanilloid receptor “hybrid” ligands. Biochemical and biophysical research communications. 2001 Feb 23;281(2):444-51.
- Di Marzo V, Bisogno T, Sugiura T, Melck D, De Petrocellis L. The novel endogenous cannabinoid 2-arachidonoylglycerol is inactivated by neuronal-and basophil-like cells: connections with anandamide. Biochemical Journal, 1998, 331.1: 15-19.
- Iannotti FA, Di Marzo V, Petrosino S (2016). Endocannabinoids and endocannabinoid‐related mediators: targets, metabolism and role in neurological disorders. Prog Lipid Res 62: 107–128.
- Esposito E, Impellizzeri D, Mazzon E, Paterniti I, Cuzzocrea S. Neuroprotective activities of palmitoylethanolamide in an animal model of Parkinson's disease. PLoS One. 2012 Aug 17;7(8):e41880.
- Facci L, Dal Toso R, Romanello S, Buriani A, Skaper SD, Leon A (1995). Mast cells express a peripheral cannabinoid receptor with differential sensitivity to anandamide and palmitoylethanolamide. Proc Natl Acad Sci U S A 92: 3376–3380.
- Ferrer I. Neurons and their dendrites in frontotemporal dementia. Dementia and geriatric cognitive disorders. 1999;10(Suppl. 1):55-60.
- Fries P. Neuronal gamma-band synchronization as a fundamental process in cortical computation. Annual review of neuroscience. 2009 Jul 21;32:209-24.
- Galasko D, Bennett DA, Sano M, Marson D, Kaye J, Edland SD. ADCS Prevention Instrument Project: assessment of instrumental activities of daily living for community-dwelling elderly individuals in dementia prevention clinical trials. Alzheimer Disease & Associated Disorders. 2006 Oct 1;20:S152-69.
- Genovese T, Esposito E, Mazzon E, Di Paola R, Meli R, Bramanti P, Piomelli D, Calignano A, Cuzzocrea S. Effects of palmitoylethanolamide on signaling pathways implicated in the development of spinal cord injury. Journal of Pharmacology and Experimental Therapeutics. 2008 Jul 1;326(1):12-23.
- Gorno-Tempini ML, Hillis AE, Weintraub S, Kertesz A, Mendez M, Cappa SE, Ogar JM, Rohrer JD, Black S, Boeve BF, Manes F. Classification of primary progressive aphasia and its variants. Neurology. 2011 Mar 15;76(11):1006-14.
- Guerreiro RJ, Lohmann E, Brás JM, Gibbs JR, Rohrer JD, Gurunlian N, Dursun B, Bilgic B, Hanagasi H, Gurvit H, Emre M. Using exome sequencing to reveal mutations in TREM2 presenting as a frontotemporal dementia–like syndrome without bone involvement. JAMA neurology. 2013 Jan 1;70(1):78-84.
- Hanajima R, Ugawa Y, Terao Y, Sakai K, Furubayashi T, Machii K, Kanazawa I. Paired‐pulse magnetic stimulation of the human motor cortex: differences among I waves. The Journal of Physiology. 1998 Jun 1;509(2):607-18.
- Heneka MT, Kummer MP, Latz E. Innate immune activation in neurodegenerative disease. Nature Reviews Immunology. 2014 Jul;14(7):463.
- Hesselink JM, Hekker TA. Therapeutic utility of palmitoylethanolamide in the treatment of neuropathic pain associated with various pathological conditions: a case series. Journal of Pain Research. 2012;5:437.
- Huang YZ, Chen RS, Rothwell JC, Wen HY. The after-effect of human theta burst stimulation is NMDA receptor dependent. Clinical Neurophysiology. 2007 May 1;118(5):1028-32.
- Huang YZ, Edwards MJ, Rounis E, Bhatia KP, Rothwell JC. Theta burst stimulation of the human motor cortex. Neuron. 2005 Jan 20;45(2):201-6.
- Hughes CP, Berg L, Danziger W, Coben LA, Martin RL. A new clinical scale for the staging of dementia. The British journal of psychiatry. 1982 Jun;140(6):566-72.
- Hughes LE, Ghosh BC, Rowe JB. Reorganisation of brain networks in frontotemporal dementia and progressive supranuclear palsy. NeuroImage: Clinical. 2013 Jan 1;2:459-68.
- Hughes LE, Rittman T, Robbins TW, Rowe JB. Reply: Brain oscillations, inhibition and social inappropriateness in frontotemporal degeneration. Brain. 2018 Sep 12;141(10):e74-.
- Ilić TV, Meintzschel F, Cleff U, Ruge D, Kessler KR, Ziemann U. Short‐interval paired‐pulse inhibition and facilitation of human motor cortex: the dimension of stimulus intensity. The Journal of physiology. 2002 Nov 1;545(1):153-67.
- Indraccolo U, Barbieri F. Effect of palmitoylethanolamide–polydatin combination on chronic pelvic pain associated with endometriosis: Preliminary observations. European Journal of Obstetrics & Gynecology and Reproductive Biology. 2010 May 1;150(1):76-9.
- Khalaj M, Saghazadeh A, Shirazi E, Shalbafan MR, Alavi K, Shooshtari MH, Laksari FY, Hosseini M, Mohammadi MR, Akhondzadeh S. Palmitoylethanolamide as adjunctive therapy for autism: Efficacy and safety results from a randomized controlled trial. J Psychiatr Res. 2018 Aug;103:104-111. doi: 10.1016/j.jpsychires.2018.04.022
- Kanazawa I, Kwak S, Sasaki H, Muramoto O, Mizutani T, Hori A, Nukina N. Studies on neurotransmitter markers of the basal ganglia in Pick's disease, with special reference to dopamine reduction. Journal of the Neurological Sciences. 1988 Jan;83(1):63-74.
- Kaufer DI, Cummings JL, Christine D. Effect of tacrine on behavioral symptoms in Alzheimer's disease: an open-label study. Journal of geriatric psychiatry and neurology. 1996 Apr;9(1):1-6.
- Kim SU, de Vellis J. Microglia in health and disease. Journal of neuroscience research. 2005 Aug 1;81(3):302-13.
- Knopman DS, Kramer JH, Boeve BF, Caselli RJ, Graff-Radford NR, Mendez MF, Miller BL, Mercaldo N. Development of methodology for conducting clinical trials in frontotemporal lobar degeneration. Brain. 2008 Oct 1;131(11):2957-68.
- Kotter MR, Zhao C, van Rooijen N, Franklin RJ. Macrophage-depletion induced impairment of experimental CNS remyelination is associated with a reduced oligodendrocyte progenitor cell response and altered growth factor expression. Neurobiology of disease. 2005 Feb 1;18(1):166-75.
- Koch G, Di Lorenzo F, Bonnì S, Ponzo V, Caltagirone C, Martorana A. Impaired LTP-but not LTD-like cortical plasticity in Alzheimer's disease patients. Journal of Alzheimer's Disease. 2012 Jan 1;31(3):593-9.
- Kujala J, Jung J, Bouvard S, Lecaignard F, Lothe A, Bouet R, Ciumas C, Ryvlin P, Jerbi K. Gamma oscillations in V1 are correlated with GABA A receptor density: A multi-modal MEG and Flumazenil-PET study. Scientific reports. 2015 Nov 17;5:16347.
- Kujirai T, Caramia MD, Rothwell JC, Day BL, Thompson PD, Ferbert A, Wroe S, Asselman P, Marsden CD. Corticocortical inhibition in human motor cortex. The Journal of physiology. 1993 Nov 1;471(1):501-19.
- Levi‐Montalcini R, Skaper SD, Dal Toso R, Petrelli L, Leon A (1996). Nerve growth factor: from neurotrophin to neurokine. Trends Neurosci 19: 514–520
- Lo Verme J, Fu J, Astarita G, La Rana G, Russo R, Calignano A et al. (2005a). The nuclear receptor peroxisome proliferator‐activated receptor‐alpha mediates the anti‐inflammatory actions of palmitoylethanolamide. Mol Pharmacol 67: 15–19
- Mann EO, Paulsen O. Role of GABAergic inhibition in hippocampal network oscillations. Trends in neurosciences. 2007 Jul 1;30(7):343-9.
- Mattace Raso G, Santoro A, Russo R, Simeoli R, Paciello O, Di Carlo C, Diano S, Calignano A, Meli R. Palmitoylethanolamide prevents metabolic alterations and restores leptin sensitivity in ovariectomized rats. Endocrinology. 2014 Apr 1;155(4):1291-301.
- Miniussi C, Thut G. Combining TMS and EEG offers new prospects in cognitive neuroscience. Brain topography. 2010 Jan 1;22(4):249.
- Palma E, Reyes-Ruiz JM, Lopergolo D, Roseti C, Bertollini C, Ruffolo G, Cifelli P, Onesti E, Limatola C, Miledi R, Inghilleri M. Acetylcholine receptors from human muscle as pharmacological targets for ALS therapy. Proc Natl Acad Sci U S A. 2016 Mar 15;113(11):3060-5.
- Petrosino S, Moriello AS, Cerrato S, Fusco M, Puigdemont A, De Petrocellis L, Di Marzo V. The anti‐inflammatory mediator palmitoylethanolamide enhances the levels of 2‐arachidonoyl‐glycerol and potentiates its actions at TRPV1 cation channels. British journal of pharmacology. 2016 Apr 1;173(7):1154-62.
- Pickford F, Marcus J, Camargo LM, Xiao Q, Graham D, Mo JR, Burkhardt M, Kulkarni V, Crispino J, Hering H, Hutton M. Progranulin is a chemoattractant for microglia and stimulates their endocytic activity. The American journal of pathology. 2011 Jan 1;178(1):284-95.
- Prinz M, Priller J. Microglia and brain macrophages in the molecular age: from origin to neuropsychiatric disease. Nature Reviews Neuroscience. 2014 May;15(5):300.
- Ranasinghe KG, Rankin KP, Lobach IV, Kramer JH, Sturm VE, Bettcher BM, Possin K, Christine You S, Lamarre AK, Shany-Ur T, Stephens ML, Perry DC, Lee SE, Miller ZA, Gorno-Tempini ML, Rosen HJ, Boxer A, Seeley WW, Rabinovici GD, Vossel KA, Miller BL. Cognition and neuropsychiatry in behavioral variant frontotemporal dementia by disease stage. Neurology. 2016 Feb 16;86(7):600-10. doi: 10.1212/WNL.0000000000002373. Epub 2016 Jan 22. PMID: 26802093; PMCID: PMC4762418.
- Rascovsky K, Hodges JR, Knopman D, Mendez MF, Kramer JH, Neuhaus J, Van Swieten JC, Seelaar H, Dopper EG, Onyike CU, Hillis AE. Sensitivity of revised diagnostic criteria for the behavioural variant of frontotemporal dementia. Brain. 2011 Aug 2;134(9):2456-77.
- Rogasch NC, Fitzgerald PB. Assessing cortical network properties using TMS–EEG. Human brain mapping. 2013 Jul;34(7):1652-69.
- Rosanova M, Casali A, Bellina V, Resta F, Mariotti M, Massimini M. Natural frequencies of human corticothalamic circuits. Journal of Neuroscience. 2009 Jun 17;29(24):7679-85.
- Ryberg E, Larsson N, Sjögren S, Hjorth S, Hermansson NO, Leonova J et al. (2007). The orphan receptor GPR55 is a novel cannabinoid receptor. Br J Pharmacol 152: 1092–1101.
- Scuderi C, Esposito G, Blasio A, Valenza M, Arietti P, Steardo Jr L, Carnuccio R, De Filippis D, Petrosino S, Iuvone T, Marzo VD. Palmitoylethanolamide counteracts reactive astrogliosis induced by β‐amyloid peptide. Journal of cellular and molecular medicine. 2011 Dec;15(12):2664-74.
- Scuderi C, Stecca C, Valenza M, Ratano P, Bronzuoli MR, Bartoli S, Steardo L, Pompili E, Fumagalli L, Campolongo P. Palmitoylethanolamide controls reactive gliosis and exerts neuroprotective functions in a rat model of Alzheimer’s disease. Cell death & disease. 2014 Sep;5(9):e1419.
- Scuderi C, Valenza M, Stecca C, Esposito G, Carratù MR, Steardo L. Palmitoylethanolamide exerts neuroprotective effects in mixed neuroglial cultures and organotypic hippocampal slices via peroxisome proliferator-activated receptor-α. Journal of neuroinflammation. 2012 Dec;9(1):49.
- Skaper SD, Facci L, Giusti P. Mast cells, glia and neuroinflammation: partners in crime? Immunology. 2014 Mar;141(3):314-27.
- Solorzano C, Zhu C, Battista N, Astarita G, Lodola A, Rivara S, Mor M, Russo R, Maccarrone M, Antonietti F, Duranti A. Selective N-acylethanolamine-hydrolyzing acid amidase inhibition reveals a key role for endogenous palmitoylethanolamide in inflammation. Proceedings of the National Academy of Sciences. 2009 Nov 18:pnas-0907417106.
- Staffaroni AM, Ljubenkov PA, Kornak J, Cobigo Y, Datta S, Marx G, Walters SM, Chiang K, Olney N, Elahi FM, Knopman DS, Dickerson BC, Boeve BF, Gorno-Tempini ML, Spina S, Grinberg LT, Seeley WW, Miller BL, Kramer JH, Boxer AL, Rosen HJ. Longitudinal multimodal imaging and clinical endpoints for frontotemporal dementia clinical trials. Brain. 2019 Feb 1;142(2):443-459. doi: 10.1093/brain/awy319. PMID: 30698757; PMCID: PMC6351779.
- Tokimura H, Di Lazzaro V, Tokimura Y, Oliviero A, Profice P, Insola A, Mazzone P, Tonali P, Rothwell JC. Short latency inhibition of human hand motor cortex by somatosensory input from the hand. The Journal of Physiology. 2000 Mar;523(2):503-13.
- Tremblay S, Rogasch NC, Premoli I, Blumberger DM, Casarotto S, Chen R, Di Lazzaro V, Farzan F, Ferrarelli F, Fitzgerald PB, Hui J. Clinical utility and prospective of TMS–EEG. Clinical Neurophysiology. 2019 Jan 19.
- Verme JL, Fu J, Astarita G, La Rana G, Russo R, Calignano A, Piomelli D. The nuclear receptor peroxisome proliferator-activated receptor-α mediates the anti-inflammatory actions of palmitoylethanolamide. Molecular pharmacology. 2005 Jan 1;67(1):15-9.
- Ziemann UL, Rothwell JC, Ridding MC. Interaction between intracortical inhibition and facilitation in human motor cortex. The Journal of physiology. 1996 Nov 1;496(3):873-81.
- Zygmunt PM, Ermund A, Movahed P, Andersson DA, Simonsen C, Jönsson BA et al. (2013). Monoacylglycerols activate TRPV1‐‐a link between phospholipase C and TRPV1. PLoS One 8 .e81618
